# Ag Nanoparticle Layer on PEDOT:PSS with Optimized Energy Levels for Improving PM6:Y6-Based Organic Photovoltaics

**DOI:** 10.1021/acsomega.5c04247

**Published:** 2025-08-15

**Authors:** Anderson E. X. Gavim, Yosthyn M. A. Florez, Patrick R. Zilz, Arandi G. Bezerra, Rafael E. de Góes, Paula C. Rodrigues, Wilson J. da Silva, Gregorio C. Faria, Paulo B. Miranda, Andreia G. Macedo, Roberto M. Faria

**Affiliations:** 1 Sao Carlos Physics Institute, University of Sao Paulo, São Carlos, SP 13566-590, Brazil; 2 CPGEI, Federal University of Technology, Curitiba, Paraná 80230-901, PR, Brazil; 3 PPGFA, Federal University of Technology, Curitiba, Paraná 80230-901, PR, Brazil; 4 PPGQ, Federal University of Technology, Curitiba, Paraná 81280-340, PR, Brazil

## Abstract

Recent advances in donor and acceptor molecules have significantly enhanced the efficiency and competitiveness of organic solar cells. However, optimizing the interfaces remains a critical issue in increasing the photovoltaic performance, mainly to reduce charge accumulation between the hole transport layers (HTLs) and the active layer. In this work, the interface between PM6:Y6 (active layer) and PEDOT:PSS (HTL) has been modified with silver nanoparticles (AgNPs). These AgNPs have been synthesized in anhydrous chlorobenzene by laser ablation synthesis in solution (LASiS). The choice of chlorobenzene as the medium for the synthesis of NPs by LASiS allows direct deposition onto the HTL. Measurements performed using steady-state current–voltage (*J*–*V*), Photo-CELIV, and current/voltage transient (TPC/TPV) revealed enhanced and reproducible photovoltaic parameters. The AgNPs also improve the device stability and can also be used on top of other HTLs, such as Br-2PACz. Theoretical analyses were performed by fitting an analytical model to the experimental data of photocurrent, which showed that the AgNP layer reduced bimolecular recombination losses. These findings suggest that the AgNP-modified interface of the PEDOT:PSS/active layer is a promising and versatile strategy to optimize interfacial properties, thus minimizing recombination losses and enhancing the efficiency, reproducibility, and stability of organic solar cells.

## Graphical abstract

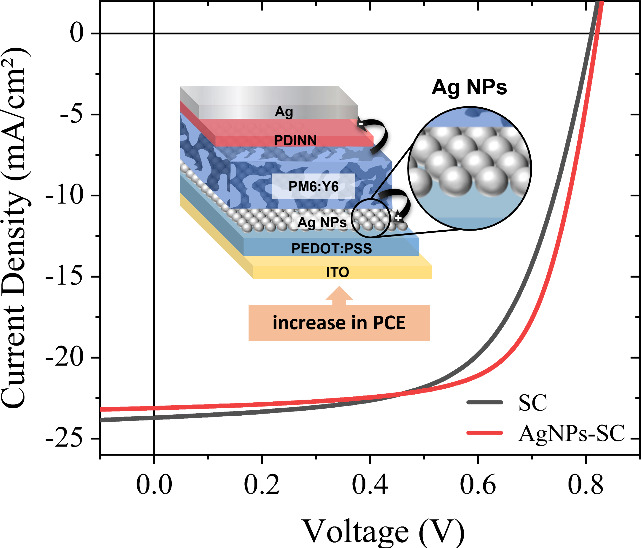

## Introduction

The emergence of novel electron donor and acceptor molecules for bulk heterojunction organic solar cells (BHJ-OSCs), particularly nonfullerene Y6-type acceptors, has significantly advanced the field by pushing power conversion efficiencies (PCE) toward 20%, making these devices increasingly competitive.^1–5^ A key breakthrough came with the efficient pairing of the PM6 donor with the Y6 acceptor, leading to substantial improvements in BHJ-OSC performance.^3^ Furthermore, incorporating aliphatic amine-functionalized perylene-diimide (PDINN) as an electron injection layer successfully down-shifted the work function of the cathodes, thereby enhancing the interfacial contact with the active layer (AL).^6^

Despite these advances, issues persist due to the multilayer structure of BHJ-OSCs, particularly those related to interfacial energy misalignment, which restrict optimal device performance.^7–9^ Additionally, the open-circuit voltage (*V*_OC_) is constrained by the disparity between quasi-Fermi energy levels of holes,^10^ which is often influenced by the alignment of energy levels at the anode interface. In an effort to address these challenges, numerous interface modifications have been explored to alter the performance and characteristics of BHJ-OSCs. Noteworthy among these efforts are hybrid graphene thin films^11,12^ and oxide/metal nanoparticles,^13,14^ which have been employed to enhance transparency, conductivity, and outdoor stability in organic photovoltaics.^15^

Thus, improving the hole-transporting layer (HTL) is critical to achieving better energy alignment and efficient charge extraction. Poly­(3,4-ethylenedioxythiophene)-poly­(styrenesulfonate) (PEDOT:PSS) remains the most widely used polymeric HTL in BHJ-OSCs, demonstrating its versatility across various organic electronic devices.^16–18^ To address the energy barriers present at the PEDOT:PSS layer interface, different approaches involving metallic nanoparticles have been applied, utilizing methods such as suspension processing,^19^ ion implantation,^20^ and aerosol techniques.^21^ A clear illustration of this was presented by Brenes-Badilla et al., who demonstrated that PEDOT:PSS degraded upon exposure to air, causing a reduction in its HOMO energy level. However, the latter was restored after gold nanoparticles that were implanted into the PEDOT:PSS film, near the HTL/AL interface.^20^ Additionally, studies have reported improvements in the PCE of organic solar cells due to the plasmonic effects generated by metal nanoparticles embedded within PEDOT:PSS.^22,23^ For example, Ganeshan et al.^24^ demonstrated that incorporating size-controlled silver nanoparticles (AgNPs) into the PEDOT:PSS layer can significantly enhance the power conversion efficiency (PCE) of fullerene-based OSCs from 7.90 to 9.45%. This was attributed to the plasmonic scattering effects of AgNPs, which increased the light absorption and charge collection at the HTL/AL interface.

In this study, the PEDOT:PSS/active layer interface has been modified with AgNPs. This procedure increased the PCE measured from ITO/PEDOT:PSS/PM6:Y6/PDINN/Ag devices. These AgNPs have been synthesized by the laser ablation synthesis in solution (LASiS) technique, where bulk metals are ablated by a laser beam in an aqueous or organic medium.^13,25^ This top-down LASiS route eliminates the precursor chemistry, ligand exchange, and high-temperature reduction steps that are inherent to wet-chemical syntheses. Such procedure yields bare metallic AgNPs that are completely surfactant-free and with their intrinsic electronic properties.^24^ Because the ablation is performed directly in anhydrous chlorobenzene, no solvent-exchange or drying sequence is required, thus avoiding water uptake by PEDOT:PSS and preventing ionic or surfactant contamination of the HTL/active-layer interface. The AgNP dispersion is dropped onto the rotating substrate and, in <1 min, forms a uniform interlayer without impacting the PEDOT:PSS layer.

This approach aimed to adjust the energy alignment at the HTL-AL interface through a straightforward deposition technique. Subsequently, a comprehensive analysis of device behavior was performed by fitting the current–voltage (*J*–*V*) characteristics using two models: (i) the traditional solar-cell equivalent-circuit equation^26^ and (ii) an analytical expression assuming second-order recombination kinetics.^27^ Ultimately, the incorporation of AgNPs also resulted in more consistent device fabrication, reducing variability in key parameters such as series and parallel resistances (*R*_s_ and *R*_sh_), fill factor (FF), and PCE.

## Experimental Section

Bulk heterojunction organic solar cells (BHJ-OSCs) are assembled layer by layer, in the following sequence: deposition of the hole transport layer (HTL) on ITO-coated glass; deposition of a nanostructured film of donor and acceptor polymers; deposition of an electron transport layer (ETL); and finally, evaporation of a metal electrode (cathode). In this work, we built two similar devices: ITO/PEDOT:PSS/PM6:Y6/PDINN/Ag, designated by SC, and ITO/PEDOT:PSS/AgNPs/PM6:Y6/PDINN/Ag, referred to as AgNPs-SC. Each step of the device manufacturing procedure (materials, the silver nanoparticle synthesis, and the device structure) is described in the Supporting Information, along with complementary characterization measurements (AFM, UV–vis spectroscopy, transient photovoltage (TPV), transient photocurrent (TPC), and Photo-CELIV). The surface potential images have been acquired by the Kelvin probe technique (Bruker Dimension Icon probe microscope): ITO (4.7 eV), PEDOT:PSS (4.95 eV), AgNPs (5.35 eV), and Ag (4.5 eV). HOMO values from the literature are PM6 (5.45 eV),^28^ Y6 (5.65 eV),^3^ and PDINN (6.02 eV).^29^

## Results and Discussion

Figure 1a presents the device geometry highlighting the localization of the AgNP layer. Figure 1b presents the energy level diagram for the OSCs built with the PEDOT:PSS/AgNPs as the HTL. The surface potential images (Figure S6) pointed out a work function of 4.94 eV for PEDOT:PSS, in accordance with values in the literature^30,31^ and the manufacturer, while the PEDOT:PSS/AgNP film presented a higher work function of 5.35 eV. Therefore, this modified HTL presents a proper energy level alignment to extract holes from the HOMO level from copolymers that have been used as donors in OSCs, such as PM6 (HOMO level ≈ 5.45 eV)^32,33^ and PTB7-Th (HOMO level ≈ 5.4 eV).^34^ Therefore, as indicated by the kelvin probe analyses, the AgNP layer improved both the interface smoothness (see also Section 3.1) and the energy alignment (Figure 1b) with the active layer, providing a lower energy barrier for the process of hole extraction. This reduces hole accumulation at the AL/HTL interface, reducing the probability of *e–h* recombination in that region.

**Figure 1:**
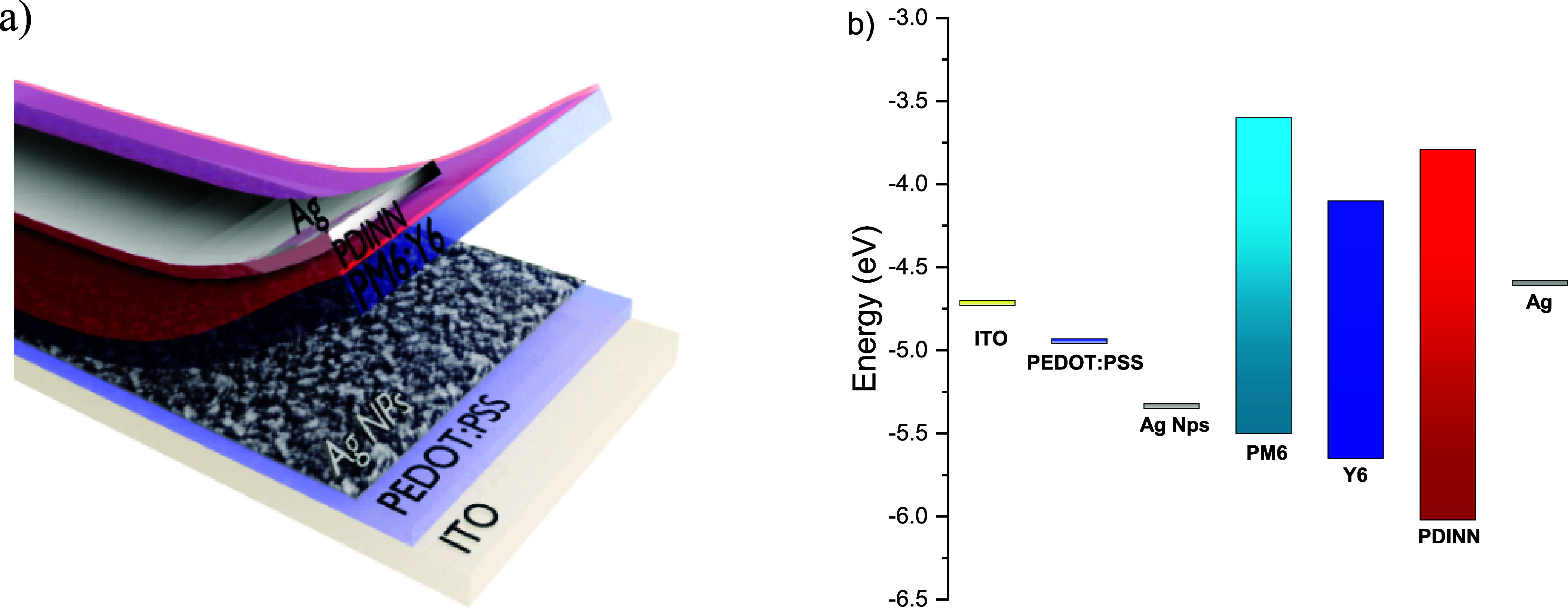
(a) Device geometry and (b) energy level diagram for devices with the structure ITO/PEDOT:PSS/AgNPs/PM6:Y6/PDINN/Ag.

### 3.1 Morphology Analysis by AFM

AFM images acquired using a high-resolution tip pointed out that the AgNPs deposited on glass in static mode (see preparation details in the Supporting Information) form aggregates with size dimensions on the order of 50 nm (Figure 2a). However, the height and phase image acquired from the films deposited by spin coating in dynamic mode (substrate upon rotation before the dropping) led to more isolated AgNPs and yielded the real (multimodal) size distribution on the order of 3, 5, and ≈12 nm (Figure 2b). Elongated structures with lengths of ca. 100 nm and diameters of ca. 10 nm have also been observed, but in minor amounts.

**Figure 2:**
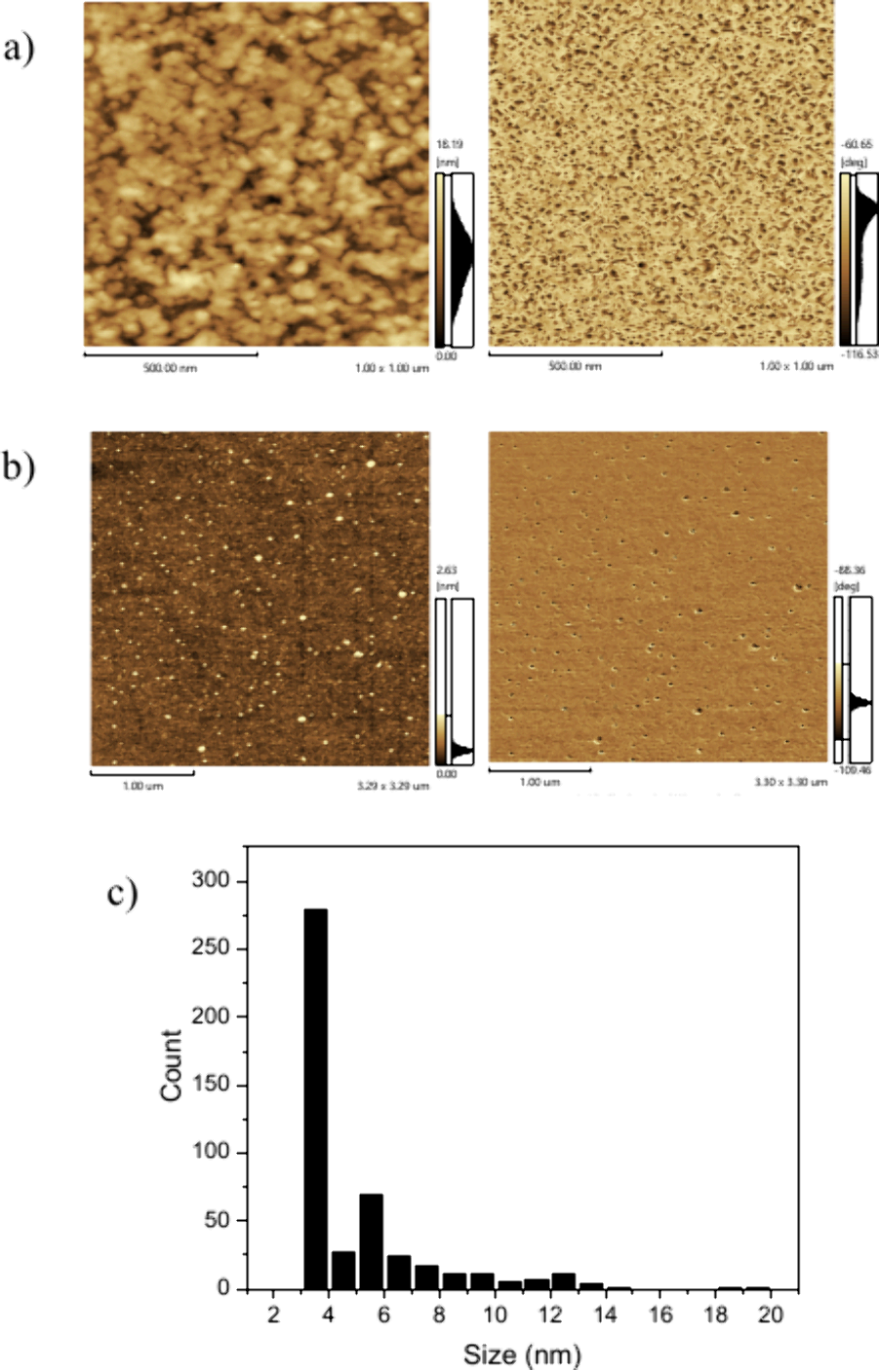
Height and phase AFM images acquired from AgNP film on glass prepared using the spin coating in (a) static mode, (b) dynamic mode, and (c) the size distribution profile from the image in (b).

The histogram (Figure 2c) reveals that particles below 12 nm dominate the population; this is compatible with dynamic light scattering (DLS) measurements of the NP suspension (Figure S3) that shows an average size of the AgNPs in suspension of ≈7.8 nm with a predominant population around 4 nm; this prevalence of smaller AgNPs should help them settle into shallow depressions on PEDOT:PSS and suppress clustering, favoring the formation of a uniform, compact overlayer. Considering that the AgNPs present a more uniform and nonaggregated distribution along the surface when the film is prepared in dynamic mode, this procedure was adopted to produce AgNP films onto the PEDOT:PSS surface.

Figure 3a–c presents the AFM height images of different batches of the pristine PEDOT:PSS film (thickness of 38.09 ± 0.99 nm, obtained by AFM, Figure S4a), which exhibits an amorphous landscape characterized by a root-mean-square roughness (Rq) of 1.51 ± 0.27 nm. In contrast, the corresponding images of the PEDOT:PSS/AgNPs (thickness of 46.16 ± 0.96 nm, obtained by AFM, Figure S4b) shown in Figure 3d–f reveals a uniform dispersion of AgNPs distributed across the polymer surface, with the roughness attenuated to 0.86 ± 0.11 nm. The ≈43% decline in Rq is probably due to the nanoparticles settling into and filling subnanometric troughs, given that chlorobenzene is not expected to appreciably swell or dissolve the underlying PEDOT:PSS matrix. Such planarization removes nanoscopic asperities that otherwise can act as charge-trapping pockets and local electric-field hot spots at the PEDOT:PSS/active-layer junction. Therefore, this smoother HTL surface, both topographically and electronically, favors more efficient carrier extraction and diminished recombination. Complementary energy-dispersive X-ray spectroscopy (EDS) elemental mapping (Figure S5) of AgNPs dispersed in a polystyrene matrix (10 wt % of AgNPs) confirms that Ag-rich regions are devoid of oxygen, demonstrating preservation of the metallic state throughout film formation (in an inert atmosphere) and handling (in air). Because LASiS produces surfactant and ligand-free AgNPs directly in anhydrous chlorobenzene, these bare particles can be deposited onto the PEDOT:PSS film without inducing polymer swelling or leaving ionic residues, concomitantly flattening the surface and up-shifting its work function from 4.95 to 5.35 eV (Figure S6), a level of interfacial optimization that traditional wet-chemical routes struggle to match.

**Figure 3:**
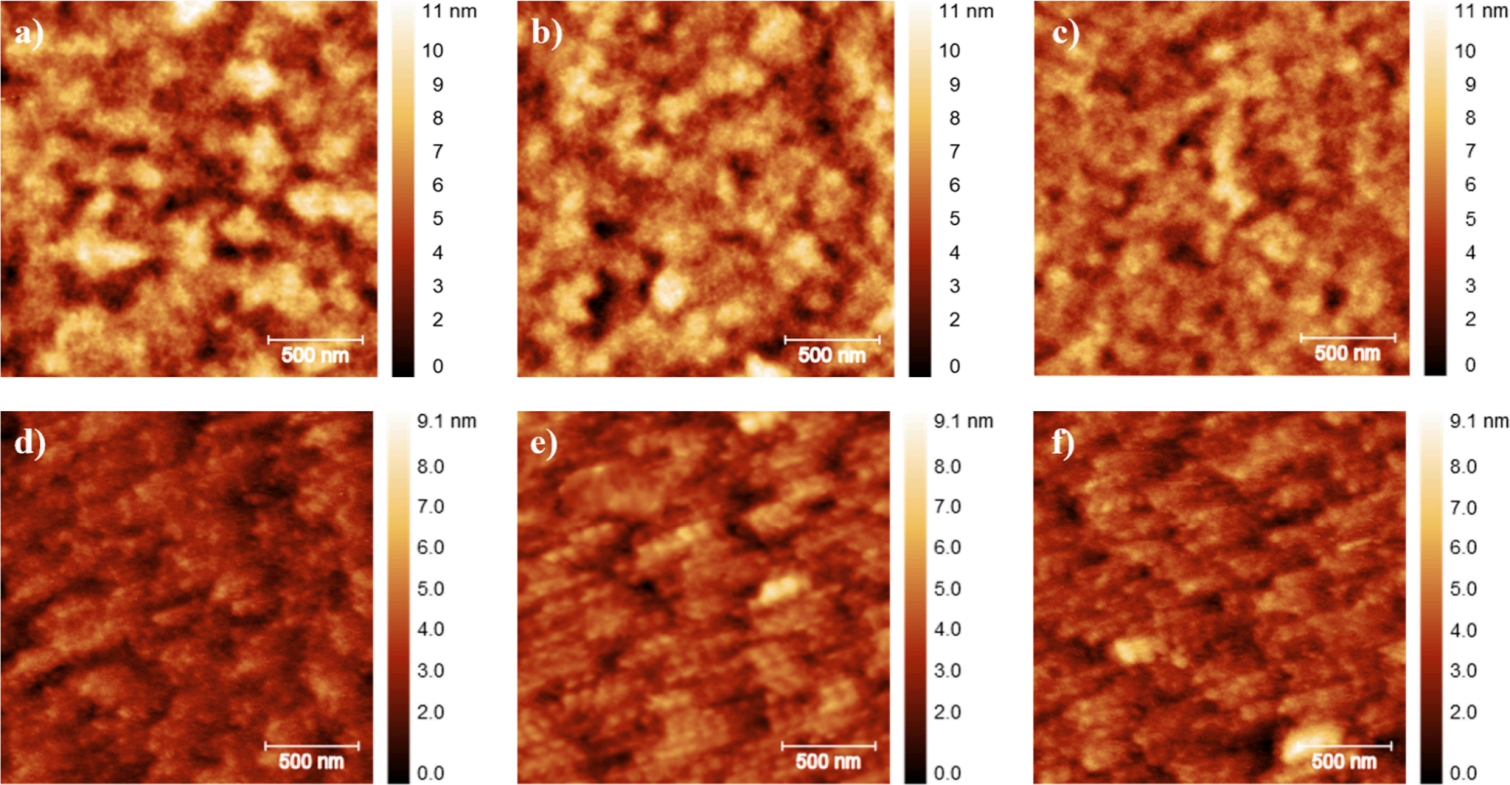
AFM height images for different batch films of PEDOT:PSS (a–c) and PEDOT:PSS/AgNPs (d–f) deposited on glass/ITO.

Moreover, Figure S7 shows the transmittance spectra acquired from the resulting films. Similar transmittance spectra have been obtained from these films, with a minor decrease at some regions, and thus, with a minor impact in the amount of light reaching the active layer with the additional AgNP layer.

### 3.2 *J–V*, TPC and TPV Transients, and Photo-CELIV Measurements

As stated by the AFM and kelvin probe analysis, the deposited layer of AgNPs on top of the PEDOT:PSS layer contributes to aligning the HOMO of the HTL with the HOMO of the active layer, which facilitates the collection of holes by the anode. To probe this, first *J*–*V* measurements of both BHJ-OSC devices, SC and AgNPs-SC, in the dark and under illumination (AM1.5G condition) are shown in Figure 4a and Figure 4b, respectively. In the dark, the diode responses of both devices are similar, and the distinction between them emerges in favor of the AgNPs-SC device only for voltages above 0.8 V, probably due to the decrease in the series resistance. The presence of AgNPs did not change the diode behavior in the region in which the *J*–*V* curve is dominated by injection of charge carriers and effects of space charge. On the other hand, under illumination, a noticeable improvement in device performance when the AgNP layer is added is shown in Figure 4b. Solar-cell parameter values, obtained from 12 devices of each type (SC and AgNPs-SC), are shown in Figure 5 and corroborate that the addition of AgNPs improves the device performance. Figure 5 also shows that the dispersions of the FF, *R*_s_, *R*_sh_, and PCE decrease considerably when depositing the nanoparticles at the PEDOT:PSS-active layer interface. This shows that nanoparticles improve reproducibility in organic solar-cell manufacturing. The average values of these parameters are shown in Table 1.

**Figure 4:**
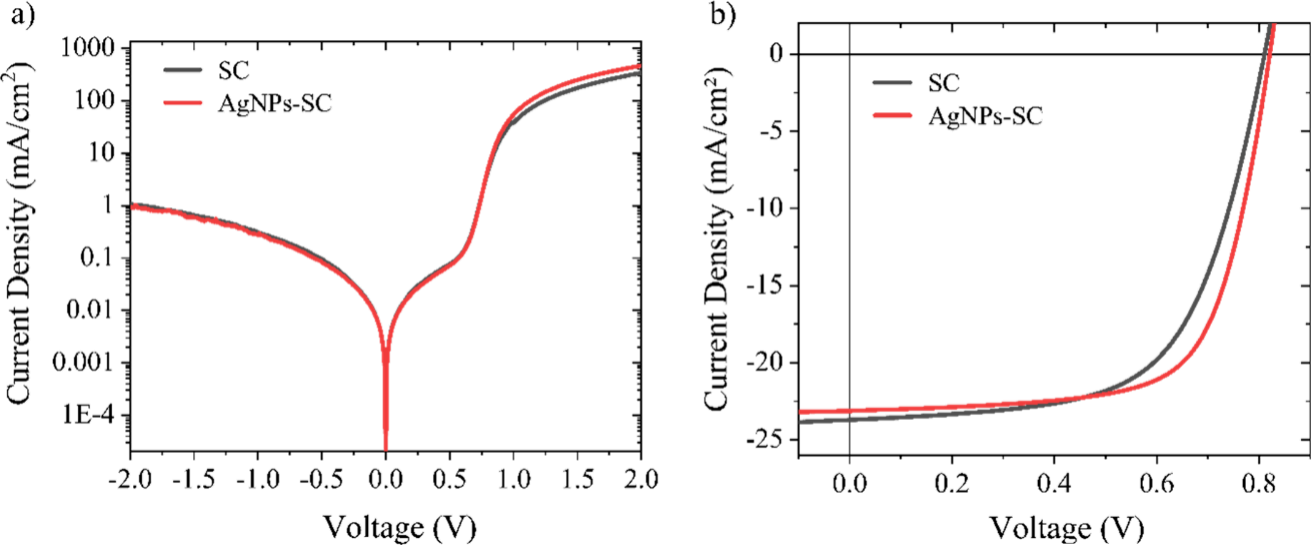
(a) *J*–*V* curves acquired in the dark, and (b) *J–V* curves acquired under illumination at the AM1.5G condition from the ITO/X/PM6:Y6/PDINN/Ag devices, where X = PEDOT:PSS or PEDOT:PSS/AgNPs.

**Figure 5:**
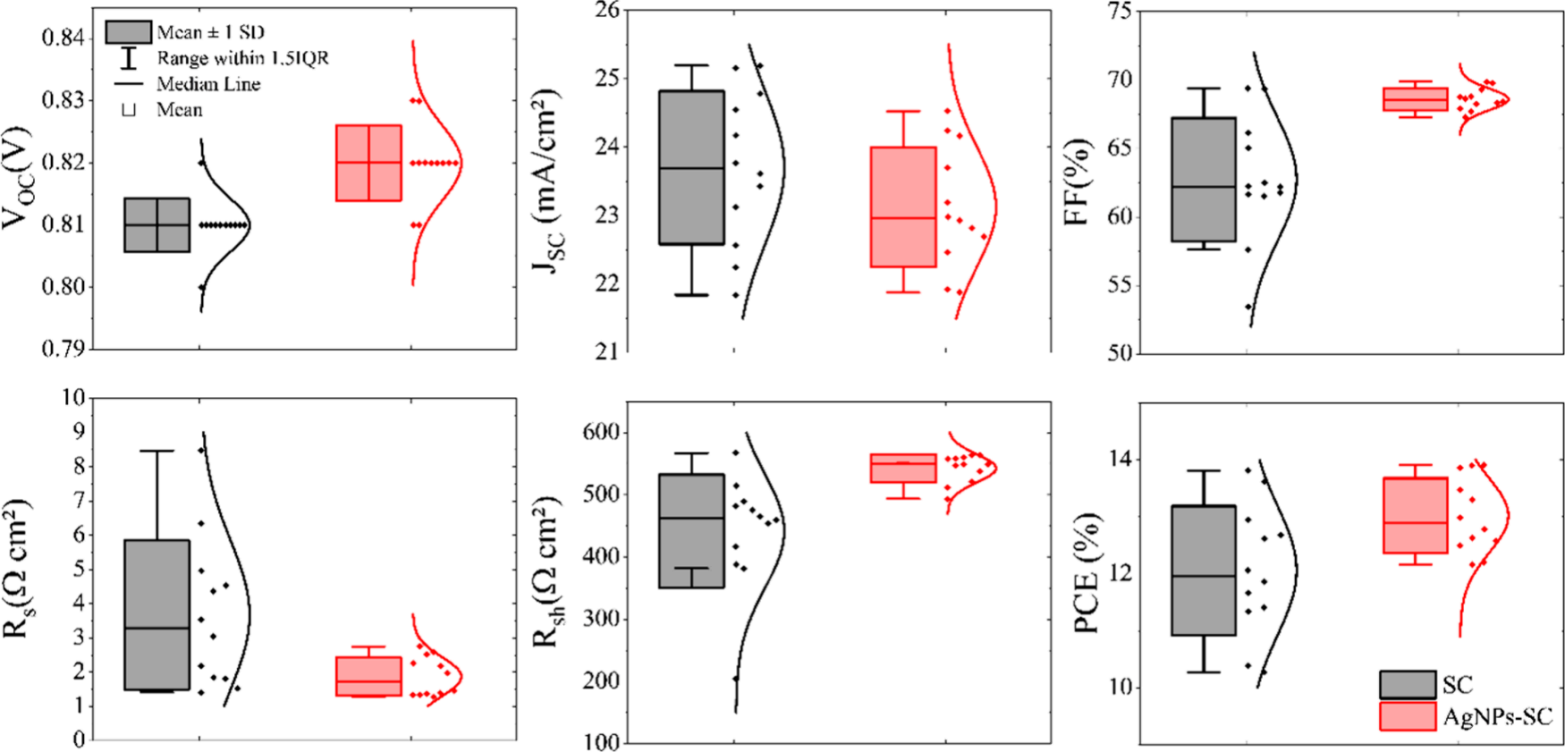
Photovoltaic parameters obtained for the ITO/X/PM6:Y6/PDINN/Ag devices, where X = PEDOT:PSS or PEDOT:PSS/AgNPs. The parameters *R*_s_ and *R*_sh_ were extracted by fitting each cell’s *J*–*V* curve with the solar-cell equivalent-circuit equation.^13^

**Table 1:** Average Photovoltaic Parameters

HTL	*V*_OC_ (V)	*J*_SC_ (mA/cm^2^)	FF (%)	*R*_s_ (Ω·cm^2^)	*R*_sh_ (Ω·cm^2^)	PCE (%)
SC	0.81	23.7	62.8	3.67	441.7	12.0
AgNPs-SC	0.82	23.1	68.6	1.87	542.7	13.0

*J*–*V* results under illumination show that the AgNPs deposited at the PEDOT:PSS-active layer interface leads to a small decrease in *J*_SC_ but contribute to a slight increase in open-circuit voltage. The slight decrease in short-circuit current is due to the equivalent decrease in absorbance of the AgNPs-SC device, as shown in Figure S7; this is further confirmed by TMM simulations of the experimental cells that shows that the simulated *J*_SC_, obtained from the exciton generation rate (*G*(*x*)) (Figure S8), decreases ≈3.5%, which is compatible with the ≈3% decrease of the experimental *J*_SC_. Because *J*_SC_ is (slightly) reduced by the deposition of AgNPs, the absorption enhancement from a possible plasmonic effect due to an evanescent field can be ruled out, since there was no significant increase in the electric current near the *J*_SC_ point. The most significant improvement occurs in the fill factor; not only the FF value shows a robust increase, but also a considerable reduction in the dispersion of values for the 12 devices measured, with respect to the devices without AgNPs. This reduction in FF dispersion for devices incorporating AgNPs is consistent with the fill factor’s relationship to the shape of the *J*–*V* curve and intrinsic device properties, such as charge mobility and recombination rate,^27^ reflecting a more reproducible device structure and fabrication. In contrast, both *J*_SC_ and the maximum extracted power remain more variable due to their heightened susceptibility to measurement conditions (e.g., fluctuations in illumination or device positioning), rather than the fabrication process itself. Similar improvement was observed in the series and parallel resistances, which shows the close relationship between these resistances and the fill factor. The significant improvement of the FF can be explained by the convergence between the HOMOs of the HTL and the active layer donor,^35^ which mitigates the accumulation of charge carriers at the interface. Additionally, it is worth emphasizing that the accumulation of charge carriers at internal interfaces of photovoltaic devices strengthens nongeminate recombination and distorts the electric field within the active layer. The ≈8% relative increase in PCE, is compatible with other works reported in literature with modifications at the HTL/Al interface for similar nonfullerene systems, such as the ≈6% relative increase in PCE for systems with PEDOT:PSS modified with sulfonated graphene^36^ and gold nano double cones.^37^ This makes the increase in PCE from ≈12 to ≈13% in devices with the AgNP layer an interesting, straightforward, and remarkable method to enhance both the PCE and reproducibility of such devices. Finally, it is worth highlighting that the efficiency of the control device is within the same range as those reported in the literature for lower molecular mass PM6,^38–40^ since the molecular mass of PM6 is an important parameter that influences the performance of high-efficiency OSCs, as shown in Figure S9 and Table S1.

Additionally, to show the versatility of the AgNP layer on top of other types of HTL to optimize the HTL/AL interface, tests were performed with Br-2PACz molecule as HTL, a known substitute to PEDOT:PSS.^41^ The Br-2PACz monolayer has a work function (5.21 eV, Figure S11b) that already matches the PM6 HOMO (5.45 eV) more closely than PEDOT:PSS (4.95 eV). After spin-coating AgNPs (5.35 eV) on Br-2PACz, the energy offset between the HTL and the HOMO of PM6 is further reduced from 0.24 to ≈0.10 eV and the rms roughness drops from 3.85 to 0.88 nm (Figure S11a). These changes translate into the performance trends shown in Figure S10, where the *J*_SC_ rises from ≈23.8 to ≈24.5 mA cm^–2^, and the average PCE increases from ≈13.5 to ≈14.2%. Because Br-2PACz already affords good energetic alignment, this smaller (≈ 5%) relative gain compared with the PEDOT:PSS case is attributed mainly to the interfacial smoothing supplied by the AgNP layer, which improves the charge collection efficiency and overcompensates the small optical loss introduced by the AgNP layer.

To further understand the charge dynamics in the cells with PEDOT:PSS, transient measurements were performed; Figure 6 shows transient photocurrent (TPC) and transient photovoltage (TPV) measurements carried out with SC and AgNPs-SC devices. The TPC decay curves of both devices showed that decay times were similar, so it can be inferred that the deposition of AgNPs into the device apparently does not influence the charge extraction mechanism. This is further confirmed by a log–log analysis of *J*_SC_ versus light intensity plot (Figure S12a) that gives α = 0.945 for SC and 0.942 for AgNPs-SC, values close to unity that confirm efficient photogeneration/extraction and negligible bimolecular losses under short-circuit conditions. TPV measurements show an increase in the decay time of the AgNPs-SC device in comparison to the SC one, indicating that the deposition of the nanoparticles increases the recombination time. This is consistent with the proposed reduction in charge accumulation at the HTL-active layer interface, which decreases recombination and leads to a higher FF. This is further confirmed by a semilog *V*_OC_ vs light intensity plot (Figure S12b) that yields slopes of 1.39 *kT*/*q* for SC and 1.22 *kT*/*q* for AgNPs-SC, evidencing a marked reduction in recombination losses when the AgNP interlayer is present. This longer TPV decay, together with the higher FF, is fully consistent with the smoother surface observed by AFM images, suggesting reduced trapping at the HTL-active layer interface; this confirms that the morphological leveling and energy alignment produced by the AgNP interlayer directly reduces interfacial recombination.

**Figure 6:**
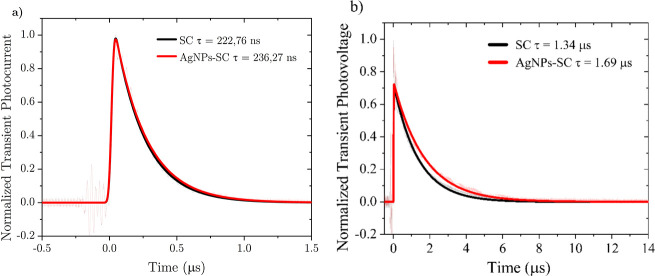
(a) TPC and (b) TPV response for ITO/X/PM6:Y6/PDINN/Ag devices, where X = PEDOT:PSS or PEDOT:PSS/AgNPs.

To further investigate the impact of AgNPs on charge extraction and mobility, Photo-CELIV measurements (Figure 7) were performed on both devices and analyzed using eq 1 from Bange et al.^42^ This equation incorporates a correction for CELIV transients, enabling the determination of mobility even under conditions of high charge density and assuming that Langevin recombination dominates. The obtained values are understood as an average value of the mobilities of electrons and holes ( $\mu =\sqrt{\mu_{e}\mu_{h}}$ ).

$$\mu =\frac{2d^{2}}{U^{\prime }t_{\max^{2}}}\left(0.860e^{-0.486\Delta j/j\left(0\right)}-0.525e^{0.0077\Delta j/j\left(0\right)}\right)$$ 1

**Figure 7:**
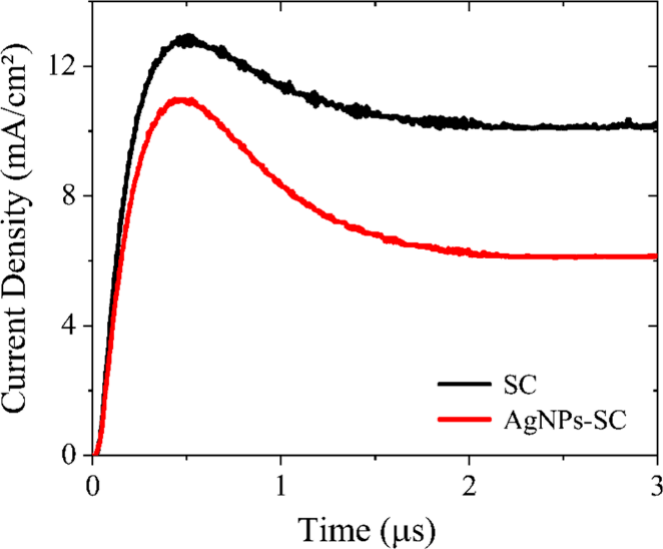
Photo-CELIV response of the ITO/X/PM6:Y6/PDINN/Ag cells, where X = PEDOT:PSS or PEDOT:PSS/AgNPs.

The calculated values for the mobilities are 4.89 × 10^–4^ and 5.12 × 10^–4^ cm^2^ V^–1^ s^–1^ for devices without and with AgNPs, respectively. This slight increase can be explained by the decrease in the charge accumulated at the HTL-active layer interface, due to the intermediate energy step interposed by the AgNPs, thus facilitating the flow of holes throughout the device and increasing the hole collection rate by the ITO electrode. That is, the enhanced hole flux between the active layer and the ITO electrode is compatible with the increased effective charge carrier mobility.

### 3.3 *J–V* Analysis by a Photocurrent Model

For a more detailed analysis of the role played by the deposition of AgNPs at the HTL-active layer interface, we adjusted the *J–V* curves under illumination by eq 2, which is a theoretical expression derived by Amorim et al.^27^ that includes the effect of second-order recombination. It is well known that in BHJ-OSCs, excitons generated by absorbed photons decay into a donor–acceptor charge transfer state (CTS), which may or may not dissociate into free charges: positive in the donor phase and negative in the acceptor phase. The recombination of a CTS pair (geminate recombination) is measured by the (1 – *P*) factor, where *P* is the probability of dissociation of CTS. After being dissociated, the photocarriers migrate toward the electrodes where they will be collected, generating the device’s electric current. However, during this migration, recombination between charge carriers originating from different CTS can occur, resulting in the called nongeminate recombination. In the Amorim model, the nongeminate recombination between the photocarriers is assumed to be of second-order kinetics, obeying a Langevin recombination mechanism. The Langevin recombination coefficient is $\gamma_{L}\left(=\frac{e\mu }{\varepsilon \varepsilon_{0}}\right)$ , and in BHJ-OSCs it is well accepted that the recombination coefficient γ is reduced relative to the Langevin coefficient by a certain factor.^43–45^

$$J(V)=\frac{2eG_{\mathrm{ct}}\mathrm{PL}}{\theta_{o}}{\left(1-\frac{V}{V_{\mathrm{bi}}}\right)}^{2}\left[\sqrt{1+\frac{\theta_{o}}{{\left(1-\frac{V}{V_{\mathrm{bi}}}\right)}^{2}}}-1\right]$$ 2

In eq 2, *G*_ct_ is the generation rate of CT states, *V*_bi_ is the built-in voltage, and θ_o_ is the physical parameter, defined by eq 3:

$$\theta_{o}=\frac{G_{\mathrm{ct}}{\mathrm{PL}}^{4}(1-P)\gamma_{L}}{(\mu V_{\mathrm{bi}}{)}^{2}}$$ 3

Figure 8a shows the fittings of SC and AgNPs-SC photocurrents extracted from Figure 4b by eq 2. However, the model ceases to be reliable for values close to the *V*_OC_, since the derivation of eq 2 does not take into account the contribution of the diffusion current, which is dominant when the device’s internal field tends to zero. For the fittings, *GP* and (1 – *P*) were used as adjustable parameters. The mobilities were obtained from the Photo-CELIV measurements, *V*_bi_ = *V*_OC_, and $\gamma_{L}\left(=\frac{e\mu }{\varepsilon \varepsilon_{0}}\right)$ is calculated using 3.5 for the dielectric constant. The values of the fitted parameters (*GP* and *P*) are exhibited in Table 2, together with θ_o_, γ = (1 – *P*)­γ_L_, and μ that were obtained from measured quantities as described above. The saturation currents calculated by equation *J*_sat_ = *eGPL*, using the *GP* values of Table 2, show excellent agreement with the values recorded in Figure 4b. Note that the largest change in those parameters occurs for the recombination rate γ, a factor of 2 lower for AgNPs-SC, consistent with our interpretation that the reduced barrier for hole extraction at the HTL-active layer interface decreases nongeminate charge recombination in these devices.

**Figure 8:**
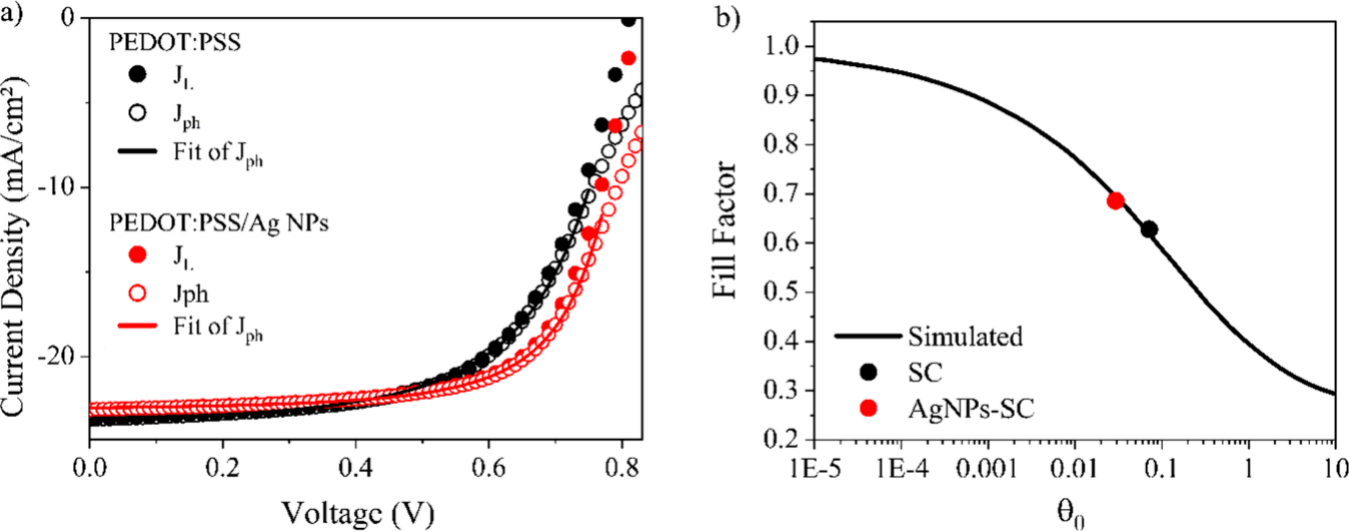
(a) Fits to eq 2 for the *J*–*V* results of Figure 4b. *J*_ph_ is the photocurrent defined by the difference between current in light condition (*J*_L_) and the current in dark condition (*J*_D_). (b) Calculated dependence of FF on the parameter θ_0_, from the Amorim model. The black and red points indicate the experimentally measured FF values for devices with PEDOT:PSS and PEDOT:PSS/AgNPs as hole transport layers.

**Table 2:** Summary of Charge Generation, Transport, and Recombination Parameters

device	θ_o_	μ (10^–4^ cm^2^ V^–1^ s^–1^)	*GP* (10^22^ cm^–3^ s^–1^)	*P*	γ (10^–11^ cm^3^ s^–1^)
SC	0.072	4.89	1.50	0.85	7.57
AgNPs-SC	0.029	5.12	1.45	0.93	3.58

The relationship that exists between the θ_o_ parameter and the FF was discussed by Bartesaghi et al., who defined θ_o_ as the ratio between the extraction rate and the recombination rate of photocarriers in BHJ-OSCs.^46^ Based on simulated *J–V* curves for a large range of FFs, they showed that FF follows a logistic type of curve (S-shaped), in which, in a monolog scale, FF is high for small values of θ_o_ and low for large values of θ_o_. In ref 46, the authors showed that results collected from 15 devices with different combinations of donor and acceptor molecules obey such FF-θ_o_ pattern. Amorim et al.^27^ have derived an FF­(θ_o_) implicit analytical expression using eq 2 and making the derivative of *J*(*V*)·*V* vanish at the maximum power point:

$$\mathrm{FF}(\theta_{o})=\frac{v(1-v{)}^{3}}{\left(2v-1)(\sqrt{1+\theta_{o}}\right)},\mathrm{with}\;\theta_{o}=\frac{\left(1-v{)}^{3}(3v-1\right)}{(2v-1{)}^{2}}$$ 4

where $v=\frac{V_{\max}}{V_{\mathrm{bi}}}$ is the voltage at the maximum power point. The “universal FF­(θ_o_) curve” is depicted in Figure 8b, and it was shown that the points of the measured FF for the SC and AgNP devices vs the respective θ_o_ values obtained from the fitting parameters are located nearly perfectly on the FF­(θ_o_) curve. This suggests that the conditions for the Amorin model (balanced electron and hole mobilities, uniform internal electric field, negligible contribution of diffusion currents, and assuming second-order recombination) are valid for our devices, at least for voltages up to the maximum power point.

### 3.4 Stability Tests

The stability of organic photovoltaic devices under atmosphere conditions is a critical parameter, particularly due to the known susceptibility of PEDOT:PSS to moisture, as it is a hygroscopic material.^47,48^ The stability of our devices was then investigated under the ISOS-T-2 protocol,^49,50^ where we subjected unencapsulated cells to 200 h of thermal cycling between 25 and 85 °C in the dark and in open air at ≈32% RH. Such conditions better approximate real-world, field-relevant stresses.

As depicted in Figure 9, AgNPs-SC demonstrates a markedly slower efficiency decay than the reference SC device, with the time required to reach 80% of the initial power conversion efficiency (*T*_80_) extending from ≈14.5 h for SC to ≈24 h for AgNPs-SC, and the 50% threshold (*T*_50_) shifting from ≈73 to ≈116 h. Both the open-circuit voltage and the *J*_SC_ are better preserved in the AgNPs-SC cells throughout the test, confirming that the metallic interlayer mitigates humidity- and temperature-driven degradation at the PEDOT:PSS/active-layer interface.

**Figure 9:**
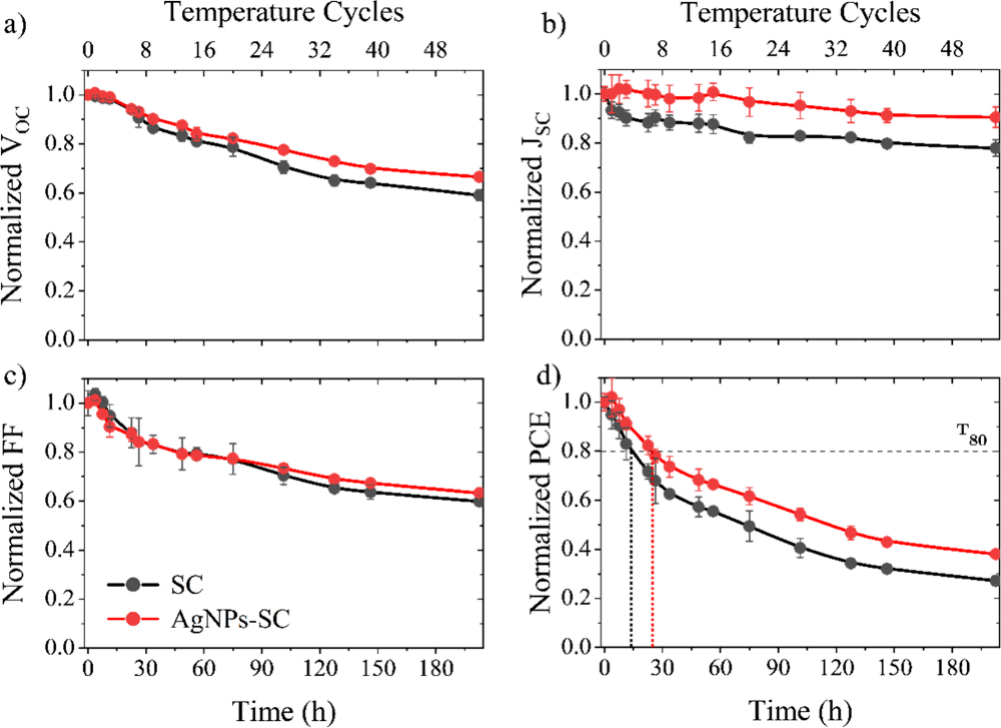
Normalized evolution of (a) *V*_OC_, (b) *J*_SC_, (c) FF, and (d) PCE for SC and AgNPs-SC devices, during the thermal cycling test (temperature ramping from 25 to 85 °C, during 3.75 h cycles, with RH of 32%) according to the ISOS-T-2 protocol.^49,50^

For comparison, a shorter but harsher high-humidity test (65–85% RH, 3 h, unencapsulated, Figure S13) likewise shows that devices containing AgNPs retain a larger fraction of their initial performance (≈46 versus ≈36% for SC), again underscoring the protective role of the nanoparticle layer under moisture exposure.

The superior performance of the AgNPs-SC can be attributed to several factors. First, the AgNP layer appears to mitigate moisture-induced degradation at the PEDOT:PSS interface, which is often a critical site for performance loss. The presence of the AgNP layer can help diminish the known moisture-assisted decohesion of the PEDOT:PSS layer at the AL interface,^51^ reducing moisture ingress and thereby preserving interfacial properties while minimizing the formation of trap states at the interface. Additionally, since *V*_OC_ is inherently dependent on the difference between the quasi-Fermi levels of the electrodes,^52^ which in turn is influenced by the energy levels of materials at the interface, and PEDOT:PSS is known to experience shifts in its work function when exposed to moisture,^53^ the AgNP layer may help delay these effects at the HTL/AL interface.

Furthermore, while metallic nanoparticles can be susceptible to oxidative processes that may decrease interfacial stability, the AgNPs employed in this study were synthesized via the LASiS technique in chlorobenzene. This method produces nanoparticles with inherently stable surface chemistry due to the absence of chemical precursors and stabilizing agents, which often contribute to oxidative degradation.^54,55^ This characteristic is likely to reduce their susceptibility to oxidation under ambient conditions and temperature stress.

Lastly, considering the strategic positioning of the AgNPs at the HTL/AL interface rather than embedded within the PEDOT:PSS bulk, the potential for significant diffusion into the layer is expected to be minimal. To verify this, cells were fabricated and stored under dark conditions in a nitrogen glovebox environment (≈3 ppm of O_2_ and moisture) for 3200 h. These conditions enable the observation of natural aging processes, including potential diffusion phenomena. Figure S14 shows the normalized photovoltaic parameters after this period for both device types, revealing that cells with and without the AgNP layer retained ≈88% of their initial PCE, which is consistent with shelf life data reported for similarly structured cells stored in the dark under an inert atmosphere.^56,57^ This result indicates no significant difference between SC and AgNPs-SC devices, and thus similar aging-related processes under these conditions, even with the presence of the AgNP layer. Overall, these findings suggest that the incorporation of the AgNP layer not only enhances device performance and moisture stability but also does not introduce additional long-term degradation pathways, supporting its viability for stable, high-performance organic photovoltaic devices.

## 4 Conclusions

This study demonstrates that the AgNP-modified PEDOT:PSS layer resulted in an enhanced photovoltaic performance from PM6:Y6-based organic photovoltaic, arising from improved energy level alignment at the HTL/active layer interface. The AgNP layer led to an 8% increase in PCE and an improved *V*_OC_ due to better HOMO alignment. TPV measurements confirmed that the AgNP layer reduces recombination losses, leading to an increased FF. Additionally, AgNPs improved the reproducibility of the devices, as reflected by reduced variability in key parameters, including FF, *R*_s_, and *R*_sh_. They also provide a protective effect against humidity and thermally induced degradation of the active layer-HTL interface, improving the device stability.

Further insights from a second-order recombination kinetics model highlighted the role of AgNPs in decreasing nongeminate recombination at the HTL-active layer interface, creating a more favorable charge transport environment. Furthermore, we have also shown that the use of AgNPs on top of the HTL is a simple and versatile strategy that can be applied to different HTL layers, as long as there is a match of the donor HOMO to the AgNPs' work function. These findings suggest that AgNPs provide an effective means of optimizing interfacial properties, which minimize recombination losses and enhance both efficiency, reproducibility and stability of organic solar cells.

## Supplementary Material



## Notes

Materials and device fabrication details, additional device characterizations, additional morphological (AFM) and spectroscopic (UV–Vis) characterizations, and basic concepts about transient measurements (PDF)

## References

[1] Li S., He C., Chen T., Zheng J., Sun R., Fang J., Chen Y., Pan Y., Yan K., Li C.-Z., Shi M., Zuo L., Ma C.-Q., Min J., Liu Y., Chen H. (2023). Refined molecular microstructure and optimized carrier management
of multicomponent organic photovoltaics toward 19.3% certified efficiency,
Energy. Environ. Sci..

[2] Fu J., Fong P. W. K., Liu H., Huang C. S., Lu X., Lu S., Abdelsamie M., Kodalle T., Sutter-Fella C. M., Yang Y., Li G. (2023). 19.31% binary
organic solar cell
and low non-radiative recombination enabled by non-monotonic intermediate
state transition. Nat. Commun..

[3] Yuan J., Zhang Y., Zhou L., Zhang G., Yip H.-L., Lau T.-K., Lu X., Zhu C., Peng H., Johnson P. A., Leclerc M., Cao Y., Ulanski J., Li Y., Zou Y. (2019). Single-Junction Organic
Solar Cell with over 15% Efficiency
Using Fused-Ring Acceptor with Electron-Deficient Core. Joule.

[4] Chen C., Wang L., Xia W., Qiu K., Guo C., Gan Z., Zhou J., Sun Y., Liu D., Li W., Wang T. (2024). Molecular interaction
induced dual fibrils towards organic solar
cells with certified efficiency over 20%. Nat.
Commun..

[5] Chen H., Huang Y., Zhang R., Mou H., Ding J., Zhou J., Wang Z., Li H., Chen W., Zhu J., Cheng Q., Gu H., Wu X., Zhang T., Wang Y., Zhu H., Xie Z., Gao F., Li Y., Li Y. (2025). Organic solar cells with 20.82% efficiency
and high
tolerance of active layer thickness through crystallization sequence
manipulation. Nat. Mater..

[6] Yao J., Qiu B., Zhang Z.-G., Xue L., Wang R., Zhang C., Chen S., Zhou Q., Sun C., Yang C., Xiao M., Meng L., Li Y. (2020). Cathode engineering
with perylene-diimide interlayer enabling over 17% efficiency single-junction
organic solar cells. Nat. Commun..

[7] Tang H., Bai Y., Zhao H., Qin X., Hu Z., Zhou C., Huang F., Cao Y. (2024). Interface
Engineering for Highly
Efficient Organic Solar Cells. Adv. Mater..

[8] Wang C., Li W., Zeng Q., Liu X., Fahlman M., Bao Q. (2023). Organic Semiconductor
Interfaces and Their Effects in Organic Solar Cells†. Chin. J. Chem..

[9] Gusain A., Faria R. M., Miranda P. B. (2019). Polymer solar cells-interfacial processes
related to performance issues. Front. Chem..

[10] Yip H.-L., Jen A. K.-Y. (2012). Recent advances
in solution-processed interfacial materials
for efficient and stable polymer solar cells, Energy. Environ. Sci..

[11] Beliatis M. J., Gandhi K. K., Rozanski L. J., Rhodes R., McCafferty L., Alenezi M. R., Alshammari A. S., Mills C. A., Jayawardena K. D. G. I., Henley S. J., Silva S. R. P. (2014). Hybrid Graphene-Metal Oxide Solution
Processed Electron Transport Layers for Large Area High-Performance
Organic Photovoltaics. Adv. Mater..

[12] Christopholi L. P., da Cunha M. R. P., Spada E. R., Gavim A. E. X., Hadano F. S., da Silva W. J., Rodrigues P. C., Macedo A. G., Faria R. M., de Deus J. F. (2020). Reduced graphene
oxide and perylene derivative nanohybrid
as multifunctional interlayer for organic solar cells. Synth. Met..

[13] Gavim A. E. X., da Cunha M. R. P., Spada E. R., Machado T. N., Hadano F. S., Ginane Bezerra A., Herwig Schreiner W., Rodrigues P. C., bin Mohd Yusoff A. R., Macedo A. G., Faria R. M., da Silva W. J. (2019). Water-suspended
MoO3 nanoparticles prepared by LASIS
and fast processing as thin film by ultrasonic spray deposition. Sol. Energy Mater. Sol. Cells.

[14] Pegg L.-J., Hatton R. A. (2012). Nanoscale Geometric Electric Field
Enhancement in Organic
Photovoltaics. ACS Nano.

[15] Kim S., Lee E., Lee Y., Kim J., Park B., Jang S.-Y., Jeong S., Oh J., Lee M. S., Kang H., Lee K. (2020). Interface Engineering
for Fabricating Semitransparent and Flexible
Window-Film-Type Organic Solar Cells. ACS Appl.
Mater. Interfaces.

[16] Santos G. H., Gavim A. A., Silva R. F., Rodrigues P. C., Kamikawachi R. C., de Deus J. F., Macedo A. G. (2016). Roll-to-roll processed
PEDOT:PSS thin films: application in flexible electrochromic devices. J. Mater. Sci.: Mater. Electron..

[17] Yamamoto N. A. D., Lima L. F., Perdomo R. E., Valaski R., Calil V. L., Macedo A. G., Cremona M., Roman L. S. (2013). Modification
of
PEDOT:PSS anode buffer layer with HFA for flexible polymer solar cells. Chem. Phys. Lett..

[18] Faria G. C., Duong D. T., Salleo A. (2017). On the transient response
of organic
electrochemical transistors. Org. Electron.

[19] Qian M., Li M., Shi X.-B., Ma H., Wang Z.-K., Liao L.-S. (2015). Planar
perovskite solar cells with 15.75% power conversion efficiency by
cathode and anode interfacial modification. J. Mater. Chem. A Mater..

[20] Brenes-Badilla D., Coutinho D. J., Amorim D. R. B., Faria R. M., Salvadori M. C. (2018). Reversing
an S-kink effect caused by interface degradation in organic solar
cells through gold ion implantation in the PEDOT:PSS layer. J. Appl. Phys..

[21] Jung K., Song H.-J., Lee G., Ko Y., Ahn K., Choi H., Kim J. Y., Ha K., Song J., Lee J.-K., Lee C., Choi M. (2014). Plasmonic Organic Solar
Cells Employing Nanobump Assembly via Aerosol-Derived Nanoparticles. ACS Nano.

[22] Ko S.-J., Choi H., Lee W., Kim T., Lee B. R., Jung J.-W., Jeong J.-R., Song M. H., Lee J. C., Woo H. Y., Kim J. Y. (2013). Highly efficient plasmonic organic
optoelectronic devices based on a conducting polymer electrode incorporated
with silver nanoparticles, Energy. Environ.
Sci..

[23] Rivera-Taco J., Castro-Beltrán R., Maldonado J.-L., Álvarez-Martínez J., Barreiro-Argüelles D., Gaspar J. A., Gutiérrez-Juárez G. (2021). The Role of
Silver Nanoparticles in the Hole Transport Layer in Organic Solar
Cells Based on PBDB-T:ITIC. J. Electron. Mater..

[24] Ganeshan D., Chen S. C., Yin Z., Zheng Q. (2015). An anode buffer layer
with size-controlled Ag nanoparticles for polymer solar cells with
improved efficiencies. RSC Adv..

[25] Rosa E. H. D. S., Gavim A. E., de Araújo F. L., de Morais A., de Freitas J. N., Bezerra A. G. Jr., Macedo A. G., da Silva W. J., Nogueira A. F. (2024). Scavenger effect of Au NPs to stabilize
the excess
of TFSI– from Spiro-OMeTAD layer. Sol.
Energy Mater. Sol. Cells.

[26] Nelson, J. A. The Physics of Solar Cells; PUBLISHED BY IMPERIAL COLLEGE PRESS AND DISTRIBUTED BY World Scientific Publishing Company: 2003. 10.1142/p276.

[27] Amorim D. R. B., Coutinho D. J., Miranda P. B., Faria R. M. (2020). Analytical Model
for Photocurrent in Organic Solar Cells as a Function of the Charge-Transport
Figure of Merit Including Second-Order Recombination. Phys. Rev. Appl..

[28] Zhang H., Yao H., Hou J., Zhu J., Zhang J., Li W., Yu R., Gao B., Zhang S., Hou J. (2018). Over 14% Efficiency
in Organic Solar Cells Enabled by Chlorinated Nonfullerene Small-Molecule
Acceptors. Adv. Mater..

[29] Zhang Z.-G., Qi B., Jin Z., Chi D., Qi Z., Li Y., Wang J. (2014). Perylene diimides:
a thickness-insensitive cathode interlayer for
high performance polymer solar cells. Energy
Environ. Sci..

[30] Cook J. H., Al-Attar H. A., Monkman A. P. (2014). Effect
of PEDOT–PSS resistivity
and work function on PLED performance. Org.
Electron.

[31] Meng Y., Hu Z., Ai N., Jiang Z., Wang J., Peng J., Cao Y. (2014). Improving the Stability of Bulk Heterojunction Solar Cells by Incorporating
pH-Neutral PEDOT:PSS as the Hole Transport Layer. ACS Appl. Mater. Interfaces.

[32] Li X., Ma R., Liu T., Xiao Y., Chai G., Lu X., Yan H., Li Y. (2020). Fine-tuning HOMO energy levels between PM6 and PBDB-T
polymer donors via ternary copolymerization. Sci. China Chem..

[33] Wang Y., Fan Q., Guo X., Li W., Guo B., Su W., Ou X., Zhang M. (2017). High-performance nonfullerene
polymer solar cells based
on a fluorinated wide bandgap copolymer with a high open-circuit voltage
of 1.04 V. J. Mater. Chem. A Mater..

[34] Khlaifia D., Alimi K. (2022). PTB7-Th /Non-fullerene
acceptors for organic solar cells. Synth. Met..

[35] Zhou X., Dong X., Liu Y., Wang W., Wei W., Chen J., Liu T., Zhou Y. (2022). Effect of Wetting Surfactants
on the Work Function of PEDOT:PSS for Organic Solar Cells. ACS Appl. Energy Mater..

[36] Pei S., Xiong X., Zhong W., Xue X., Zhang M., Hao T., Zhang Y., Liu F., Zhu L. (2022). Highly Efficient Organic
Solar Cells Enabled by the Incorporation of a Sulfonated Graphene
Doped PEDOT:PSS Interlayer. ACS Appl. Mater.
Interfaces.

[37] Zhao Y., Wu Y., Song J., Zeng J., Dai Z., Li H., Quan J., Zong Q., Liang G., Lin B., Zheng Z., Guo L., Liu S., Wang H., Zhou E., Li Z. (2025). An effective universal ternary complex
hole transport material enabled by intermolecular interactions for
polymer solar cells. Colloids Surf. A Physicochem
Eng. Asp.

[38] Adil M. A., Memon W. A., Zhang J., Iqbal M. J., Yang C., Wang Y., Zou W., Wei Z. (2022). Utilizing Ternary Strategy
to Reduce the Influence of Polymer Batch-to-Batch Variation in Organic
Solar Cells. Sol. RRL.

[39] Yoon S., Schopp N., Choi D. G., Wakidi H., Ding K., Ade H., Vezin H., Reddy G. N. M., Nguyen T. Q. (2024). Influences of Metal
Electrodes on Stability of Non-Fullerene Acceptor-Based Organic Photovoltaics. Adv. Funct. Mater..

[40] Yoon S., Reyes-Suárez B., Pham S. T., Vezin H., Tobon Y. A., Lee M., Mugiraneza S., Kim B. M., Oide M. Y. T., Yoo S., Lee S., Wang S. H., Collins S. M., Bates C. M., Park Y., Kim B. S., Manjunatha Reddy G. N., Nguyen T. Q. (2025). Molecular Cross-Linking
Enhances Stability of Non-Fullerene Acceptor Organic Photovoltaics. ACS Energy Lett..

[41] Lin Y., Magomedov A., Firdaus Y., Kaltsas D., El-Labban A., Faber H., Naphade D. R., Yengel E., Zheng X., Yarali E., Chaturvedi N., Loganathan K., Gkeka D., AlShammari S. H., Bakr O. M., Laquai F., Tsetseris L., Getautis V., Anthopoulos T. D. (2021). 18.4% Organic
Solar Cells Using a High Ionization Energy Self-Assembled Monolayer
as Hole-Extraction Interlayer. ChemSusChem.

[42] Bange S., Schubert M., Neher D. (2010). Charge mobility
determination by
current extraction under linear increasing voltages: Case of nonequilibrium
charges and field-dependent mobilities. Phys.
Rev. B: Condens. Matter Mater. Phys..

[43] Lakhwani G., Rao A., Friend R. H. (2014). Bimolecular
Recombination in Organic Photovoltaics. Annu.
Rev. Phys. Chem..

[44] Burke T. M., Sweetnam S., Vandewal K., McGehee M. D. (2015). Beyond Langevin
Recombination: How Equilibrium Between Free Carriers and Charge Transfer
States Determines the Open-Circuit Voltage of Organic Solar Cells. Adv. Energy Mater..

[45] Liu Y., Zojer K., Lassen B., Kjelstrup-Hansen J., Rubahn H.-G., Madsen M. (2015). Role of the Charge-Transfer State
in Reduced Langevin Recombination in Organic Solar Cells: A Theoretical
Study. J. Phys. Chem. C.

[46] Bartesaghi D., Pérez I. D. C., Kniepert J., Roland S., Turbiez M., Neher D., Koster L. J. A. (2015). Competition between recombination
and extraction of free charges determines the fill factor of organic
solar cells. Nat. Commun..

[47] Liu L., Wu L., Yang H., Ge H., Xie J., Cao K., Cheng G., Chen S. (2022). Conductivity
and Stability Enhancement
of PEDOT:PSS Electrodes via Facile Doping of Sodium 3-Methylsalicylate
for Highly Efficient Flexible Organic Light-Emitting Diodes. ACS Appl. Mater. Interfaces.

[48] Xu B., Gopalan S. A., Gopalan A. I., Muthuchamy N., Lee K. P., Lee J. S., Jiang Y., Lee S. W., Kim S. W., Kim J. S., Jeong H. M., Kwon J. B., Bae J. H., Kang S. W. (2017). Functional solid
additive modified
PEDOT:PSS as an anode buffer layer for enhanced photovoltaic performance
and stability in polymer solar cells. Sci. Rep..

[49] Reese M. O., Gevorgyan S. A., Jo̷rgensen M., Bundgaard E., Kurtz S. R., Ginley D. S., Olson D. C., Lloyd M. T., Morvillo P., Katz E. A., Elschner A., Haillant O., Currier T. R., Shrotriya V., Hermenau M., Riede M., Kirov K. R., Trimmel G., Rath T., Inganäs O., Zhang F., Andersson M., Tvingstedt K., Lira-Cantu M., Laird D., McGuiness C., Gowrisanker S., Pannone M., Xiao M., Hauch J., Steim R., DeLongchamp D. M., Rösch R., Hoppe H., Espinosa N., Urbina A., Yaman-Uzunoglu G., Bonekamp J.-B., van Breemen A. J. J.
M., Girotto C., Voroshazi E., Krebs F. C. (2011). Consensus stability testing protocols
for organic photovoltaic materials and devices. Sol. Energy Mater. Sol. Cells.

[50] Florez, Y. M. A. Células solares de terceira geração: estudo do efeito da degradação nos parâmetros fotovoltaicos. Doctoral dissertation, Universidade de São Paulo, 2023. 10.11606/D.76.2023.tde-02012024-110204.

[51] Dupont S. R., Novoa F., Voroshazi E., Dauskardt R. H. (2014). Decohesion
kinetics of PEDOT:PSS conducting polymer films. Adv. Funct Mater..

[52] Qi B., Wang J. (2012). Open-circuit voltage in organic solar cells. J. Mater. Chem..

[53] Wachsmuth J., Distler A., Deribew D., Salvador M., Brabec C. J., Egelhaaf H. J. (2023). Overcoming Moisture-Induced Degradation in Organic
Solar Cells. Adv. Eng. Mater..

[54] Scaramuzza S., Agnoli S., Amendola V. (2015). Metastable
alloy nanoparticles, metal-oxide
nanocrescents and nanoshells generated by laser ablation in liquid
solution: Influence of the chemical environment on structure and composition. Phys. Chem. Chem. Phys..

[55] Amendola V., Meneghetti M. (2009). Laser ablation synthesis in solution and size manipulation
of noble metal nanoparticles. Phys. Chem. Chem.
Phys..

[56] Ghasemi M., Balar N., Peng Z., Hu H., Qin Y., Kim T., Rech J. J., Bidwell M., Mask W., McCulloch I., You W., Amassian A., Risko C., O’Connor B. T., Ade H. (2021). A molecular interaction–diffusion
framework for predicting
organic solar cell stability. Nat. Mater..

[57] Tu S., Lin X., Xiao L., Zhen H., Wang W., Ling Q. (2022). Boosting the
overall stability of organic solar cells by crosslinking vinyl-functionalized
polymer derived from PM6. Mater. Chem. Front.

