# Supplementary material for: Ag Nanoparticle Layer on PEDOT:PSS with Optimized Energy Levels for Improving PM6:Y6-Based Organic Photovoltaics
Source: ACS Omega. 2025 Aug 15;10(33):37664–73. doi: 10.1021/acsomega.5c04247 (PMC12391953; doi:10.1021/acsomega.5c04247)
Supplement: Supplementary file 1 [file ao5c04247_si_001.pdf]

Ag Nanoparticle Layer on PEDOT:PSS with Optimized Energy Levels for Improving PM6:Y6-Based Organic Photovoltaics

Anderson E. X. Gavim[1], Yosthyn M. A. Florez[1], Patrick R. Zilz[2], Arandi G. Bezerra Jr[3*], Rafael E. de Góes[3], Paula C. Rodrigues[4], Wilson J. da Silva[2], Gregorio C. Faria[1], Paulo B. Miranda[1], Andreia G. Macedo[3], Roberto M. Faria[1,3,4*]

[1] Sao Carlos Physics Institute, University of Sao Paulo, São Carlos 13566-590, SP, Brazil

[2]CPGEI, Federal University of Technology - Paraná, Curitiba 80230-901, PR, Brazil

[3]PPGFA, Federal University of Technology - Paraná, Curitiba 80230-901, PR, Brazil

[4]PPGQ, Federal University of Technology - Paraná, Curitiba 81280-340, PR, Brazil

[*]Email: faria@ifsc.usp.br, arandi@utfpr.edu.br

Supplementary Information

S1 – Materials and *devices fabrication*

ITO (8-12 Ω/sqr, Delta), PEDOT:PSS (Clevios P AI 4083, HERAEUS), Poly[[4,8-bis[5-(2-ethylhexyl)-4-fluoro-2-thienyl]benzo-[1,2-b:4,5-b']dithiophene-2,6-diyl]-2,5-thiophenediyl-[5,7-bis(2-ethylhexyl)-4,8-dioxo-4H,8H-benzo[1,2-c:4,5-c']-dithiophene-1,3-diyl]-2,5-thiophenediyl] (PM6, LUMTEC), 2,2'-((2Z,2'Z)-((12,13-Bis(2-ethylhexyl)-3,9-diundecyl-12,13-dihydro-[1,2,5]thiadiazolo[3,4-e]thieno-[2",3":4',5']thieno[2',3':4,5]pyrrolo[3,2-g]thieno-[2',3':4,5]thieno[3,2-b]indole-2,10 diyl)bis(methanylylidene))-bis(5,6-difluoro-3-oxo-2,3-dihydro-1H-indene-2,1-diylidene))dimalononitrile (Y6, LUMTEC), N,N'-Bis{3-[3-(dimethylamino)propylamino]propyl}perylene-3,4,9,10-tetracarboxylic diimide (PDINN, LUMTEC), Poly(9,9-bis(3'-(*N*,*N*-dimethyl)-*N*-ethylammoinium-propyl-2,7-fluorene)-alt-2,7-(9,9-dioctylfluorene))dibromide (PFN-Br, LUMTEC), (2-(3,6-Dibromo-9H-carbazol-9-yl)ethyl)phosphonic acid (Br-2PACz, LUMTEC). The anhydrous chlorobenzene (CB), chloroform (CF), and methanol (MeOH) were purchased from Aldrich and used without further purification.

Synthesis of AgNPs by LASiS: Synthesis of Ag NPs by LASiS: Ag NPs were prepared using a procedure reported in the reference [12] of the main paper, where the Ag target was immersed in anhydrous CB (15 mL) and irradiated by a pulsed laser (1064nm, 50kHz, 400ns, 0,5mJ) with a 20 cm focusing lens, for 5 min. Figure S1 shows a picture of AgNPs in CB suspension.

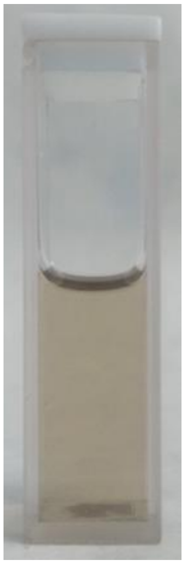

Figure S1 - Image of the AgNPs in CB suspension.

OSCs devices fabrication: glass/ITO substrates were sequentially cleaned in an ultrasonic bath in Extran, ultrapure water, acetone, and isopropyl alcohol, (15 min each step). These substrates were further exposed to UV-ozone for 15 minutes. Then, for the cells that used PEDOT:PSS as HTL it was deposited by spin coating at 3000 rpm for 60 s, resulting in a thin film of 30 ± 0.5 nm, followed by a thermal annealing at 120 °C for 10 min. For the cells that used Br-2PACz as the HTL, a solution of 0.3 mg/ml in ethanol was deposited by spin coating at 3000 rpm for 15 s, followed by thermal annealing at 100 °C for 5 min. This was followed by two subsequent spin-coating washes at 6000 rpm for 20 s each using the Br-2PACz solution. Subsequently, a volume of 150 µL of AgNPs suspension in CB was deposited onto the PEDOT:PSS layer upon rotation at 2000 rpm for 60 s (dynamic mode) resulting in a film with thickness of ~10 nm. Afterwards, the BHJ active layer composed of PM6:Y6 (1:1.2, 16 mg/mL in chloroform) was deposited onto the glass/ITO/PEDOT:PSS/AgNPs by spin coating at 3000 rpm for 60 s. The resulting thickness was 100 ± 0.3 nm. The PDINN ETL film was deposited from methanol solution (1 mg/mL) at 3000 rpm for 40 s, resulting in a thin film with a thickness of ~ 5 nm. Finally, the Ag anode (100 nm) was deposited by thermal evaporation at $1x10^{-6}$ mBar, with an evaporation rate of 0.5 Å/s for the first 10 nm and 2 Å/s for the rest of the process, yielding devices with an active area of 4.5 mm^2. The device response was evaluated by JxV curves acquired using a Keithley 2400 electrometer upon illumination of 100 mW/cm^2 from a Solar Simulator (Oriel Class AAA with an AM 1.5G filter).

S2 - Material characterization

UV-Vis spectra were acquired from AgNPs in CB suspension placed in a 1 cm pathlength quartz cuvette and from AgNPs films deposited onto quartz substrate, in a Hitachi U-2900 spectrophotometer at the wavelength range of 200-1100 nm with a step of 2 nm. An absorbance curve of a AgNPs film in is shown in Fig. S2. In the inset is shown the absorbance of AgNPs in CB emulsion. Fig. S3 compares the optical transmission of the PEDOT:PSS film with and without the additional AgNPs layer on top of it.

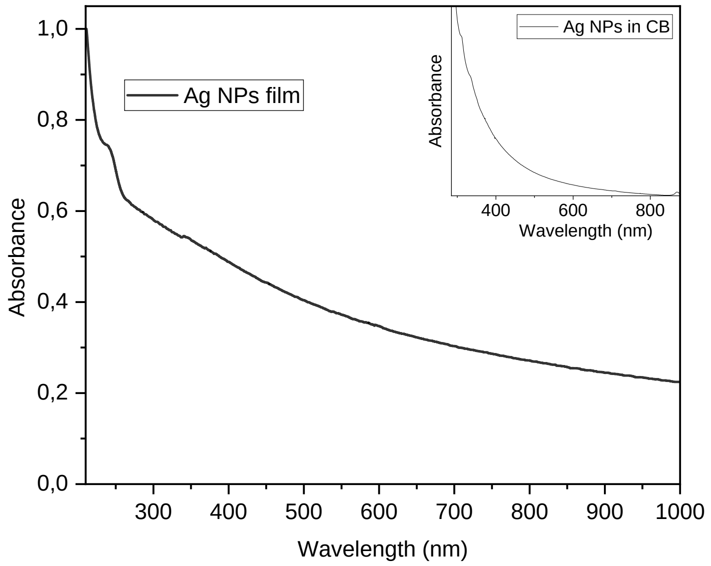

Figure S2 - Absorbance spectrum of Ag NPs film deposited by drop casting method onto a quartz substrate(~ 100 nm thickness). Inset: the absorbance spectrum acquired from Ag NPs in CB suspension.

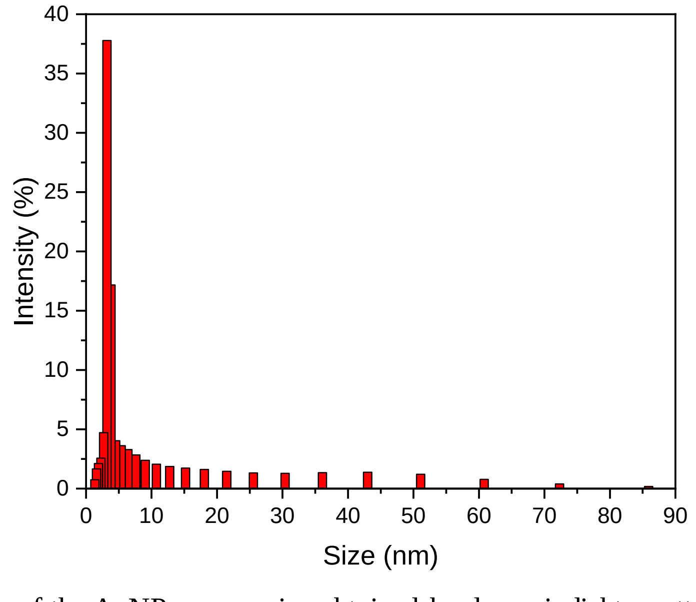

Figure S3 –Size distribution of the AgNPs suspension obtained by dynamic light scattering (DLS).

For the AFM analyses in Section 3.1, AgNPs were processed as thin films by the spin coating method. About 100 µL of the AgNPs suspension was dropped onto the substrate in static or dynamic mode. In static mode, the suspension was dropped onto the substrate, then it started to rotate until achieving 2000 rpm, with an acceleration rate of 400 rpm/s. In dynamic mode, the substrate was rotating at 2000 rpm when the suspension was dropped. In both cases, the film remains spinning at 2000 rpm for 1 minute, after the deposition. Then, the resulting films were analyzed in tapping mode by an AFM microscope model SPM-9700 HT from Shimadzu, using a high-resolution AFM probe (SHR300, Budget Sensors, force constant 40 N/m, resonance frequency 300 kHz, gold overall coating, DLC spike not coated). The size distribution histogram was obtained by using the "particle analyses" resource available in the NanoMapping 3D software from Shimadzu. Glass/ITO/PEDOT:PSS and glass/ITO/PEDOT:PSS/AgNPs were analyzed in a Nanoscope III AFM using a SiN_3 probe in tapping mode. The surface potential (Fig. S4) was acquired while using pyrolytic graphite as a reference.

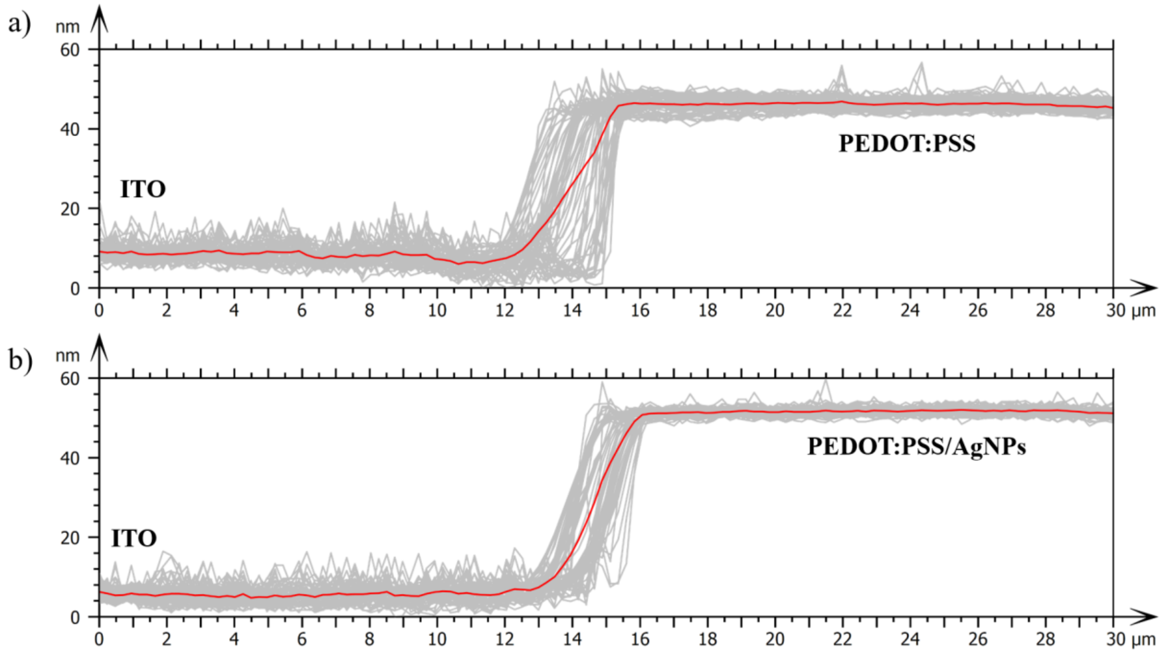

Figure S4 - Side-view height profiles of the a) ITO/PEDOT:PSS and b) ITO/PEDOT:PSS/AgNPs samples extracted from AFM topography images. Grey traces show the 128 individual line scans recorded for each configuration, and the red solid line represents their mean profile.

Energy-dispersive X-ray spectroscopy (EDS) elemental mapping was carried out on a field-emission scanning electron microscope (FE-SEM, JEOL JSM-IT500HR) operated at an accelerating voltage of 10 kV. The microscope is equipped with a EDS detector (JEOL EX-74212U4L2Q).

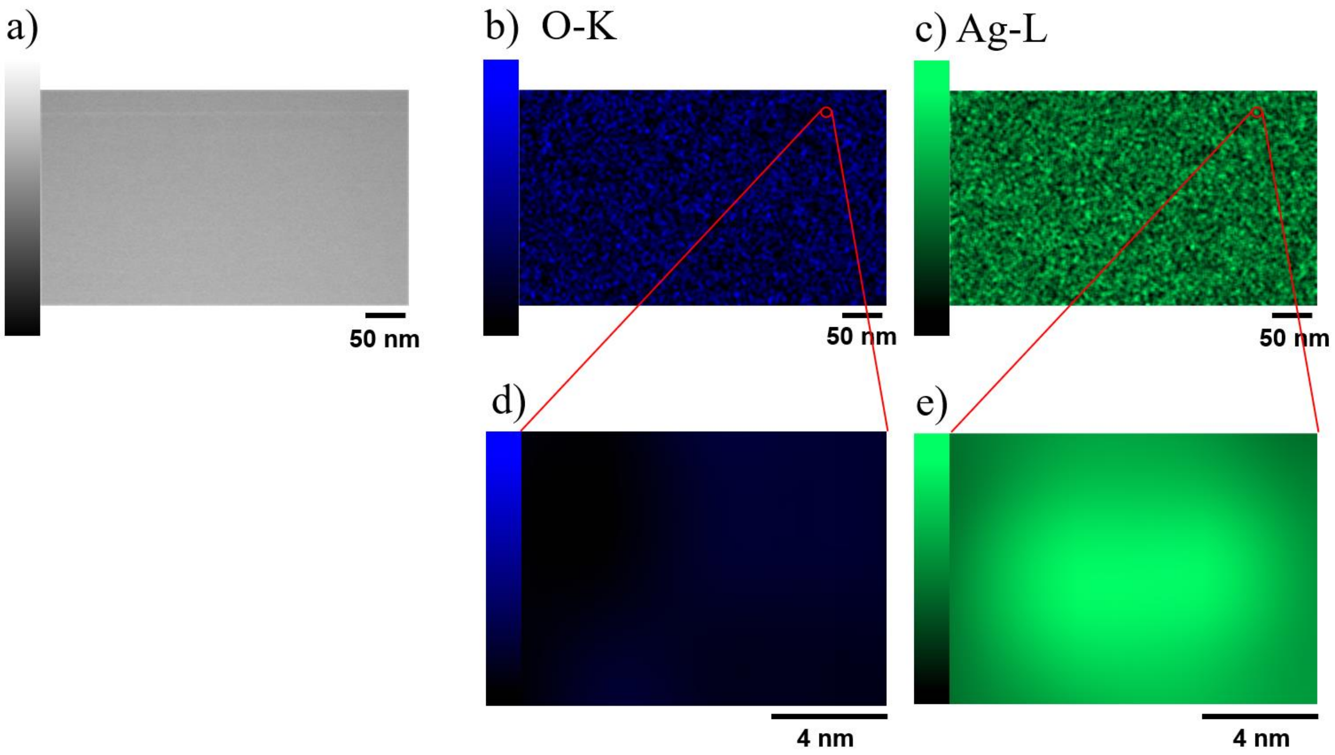

Figure S5 - a) SEM micrograph of the AgNPs/polystyrene (10 wt %) film and the corresponding EDS elemental maps of b) oxygen and c) silver for the same area. (d, e) Enlarged views of the region marked by the red circle.

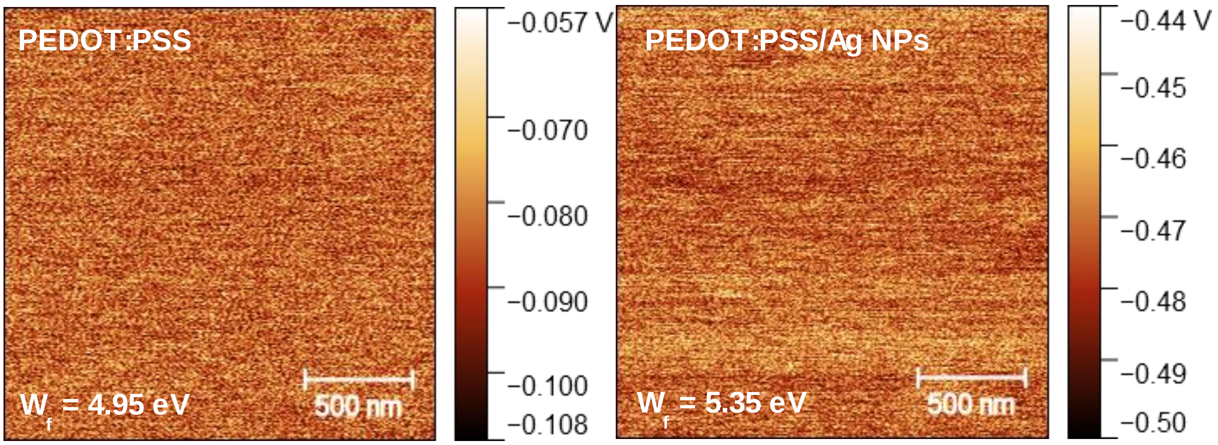

Figure S6 - Surface potential images obtained from ITO/PEDOT:PSS and ITO/PEDOT:PSS/Ag NPs thin films by using the Kelvin Probe method.

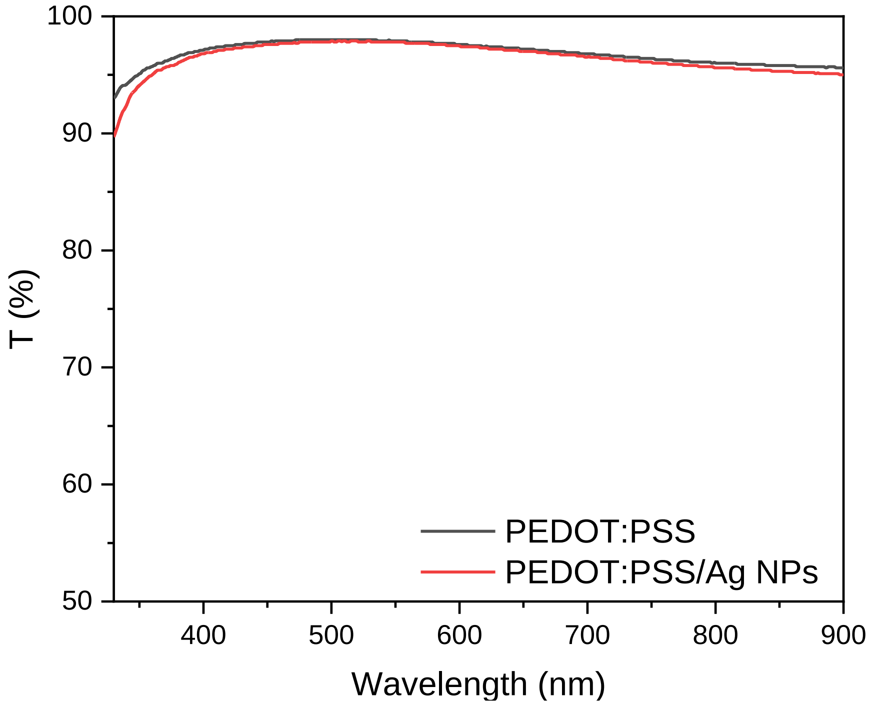

Figure S7 - UV-VIS transmittance spectra of PEDOT:PSS and PEDOT:PSS/AgNPs.

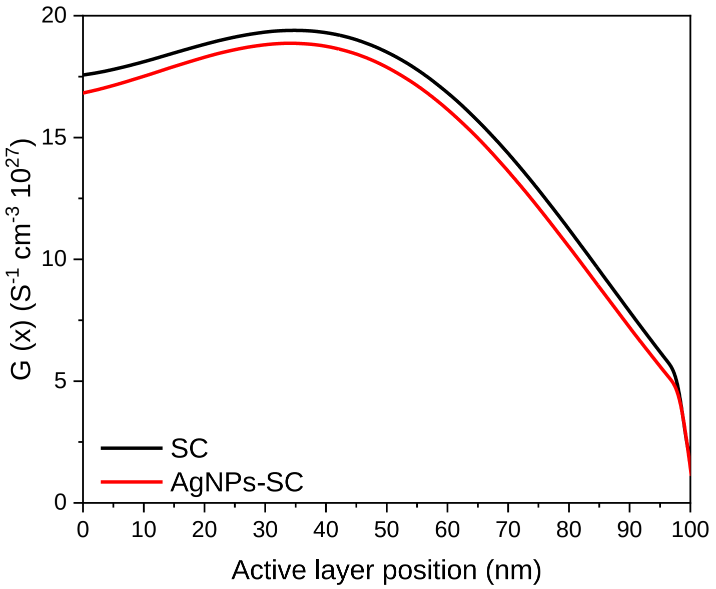

Figure S8 - Exciton-generation profile G(x) calculated by transfer-matrix modelling (TMM) for the stack ITO (130 nm)/X/PM6:Y6 (100nm)/PFN-Br (5 nm)/Ag (100 nm), where X = PEDOT:PSS (38 nm) either without or with an 8 nm AgNPs interlayer. The absorption coefficient (α) of the AgNPs film was extracted from its absorbance spectrum, and the corresponding extinction coefficient (k) was derived from transmittance spectra following the procedure detailed in reference [1]. The simulations were performed with the OghmaNano software [2].

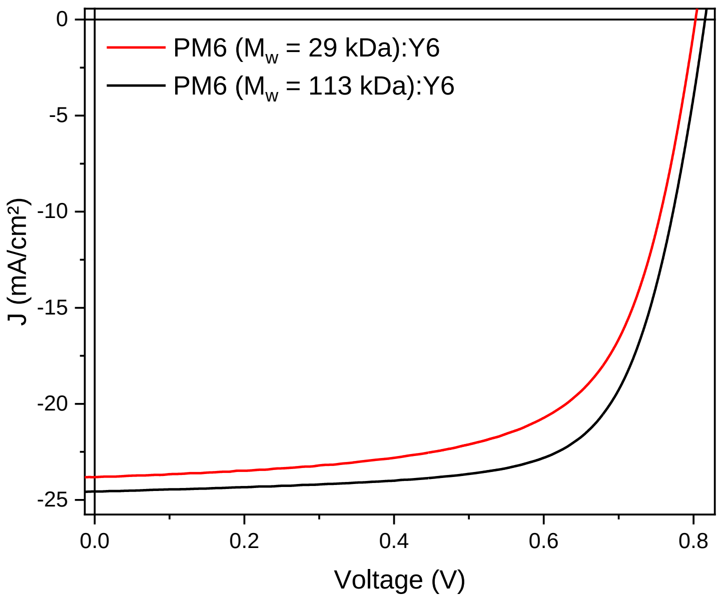

Figure S9 - *J-V* curves acquired under illumination at the AM1.5G condition for the ITO (130 nm)/PEDOT:PSS (30 nm)/ PM6:Y6 (100 nm)/ PFN-Br (5nm)/Ag (120nm) devices. The plot compares the performance of PM6 used in this work (provided by LUMTEC, GPC M_w = 29 kDa) and other PM6 with higher Molecular Mass (Mw) (provided by Solarmer, GPC M_w = 113 kDa).

Table S1 - Summarized Photovoltaic parameters of OSCs with the structure ITO (130 nm)/PEDOT:PSS (30 nm)/ PM6:Y6 (100 nm)/ PFN-Br (5nm)/Ag (120nm):

**Donor**	**M_w (kDa)**	**V_{oc} (V)**	**J_{sc} (mA/cm^2)**	**FF (%)**	**PCE (%) (Av. and Best)**
**PM6**	29	0.803 ± 0.01	23.82 ± 0.87	66.02 ± 0.70	12.62 ± 0.42 (13.214)
**PM6**	113	0.815 ± 0.01	24.55 ± 0.98	70.60 ± 0.67	14.14 ± 0.63 (15.21)

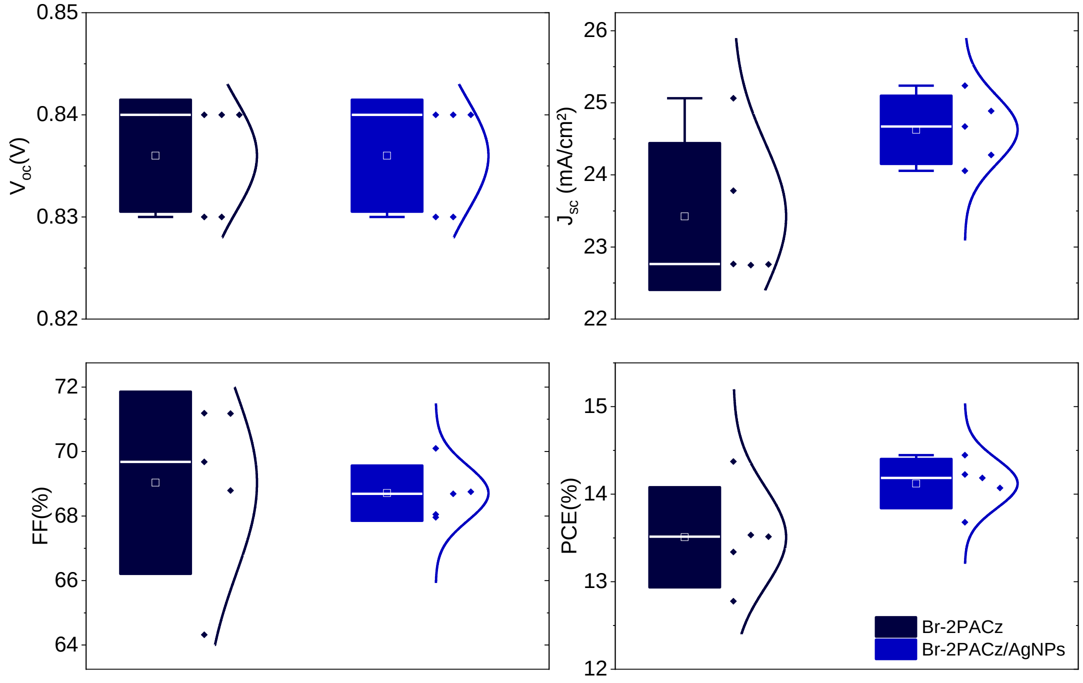

Figure S10 - Photovoltaic parameters obtained for the ITO/X/PM6:Y6/PDINN/Ag devices, where X= Br-2PACz or Br-2PACz/AgNPs.

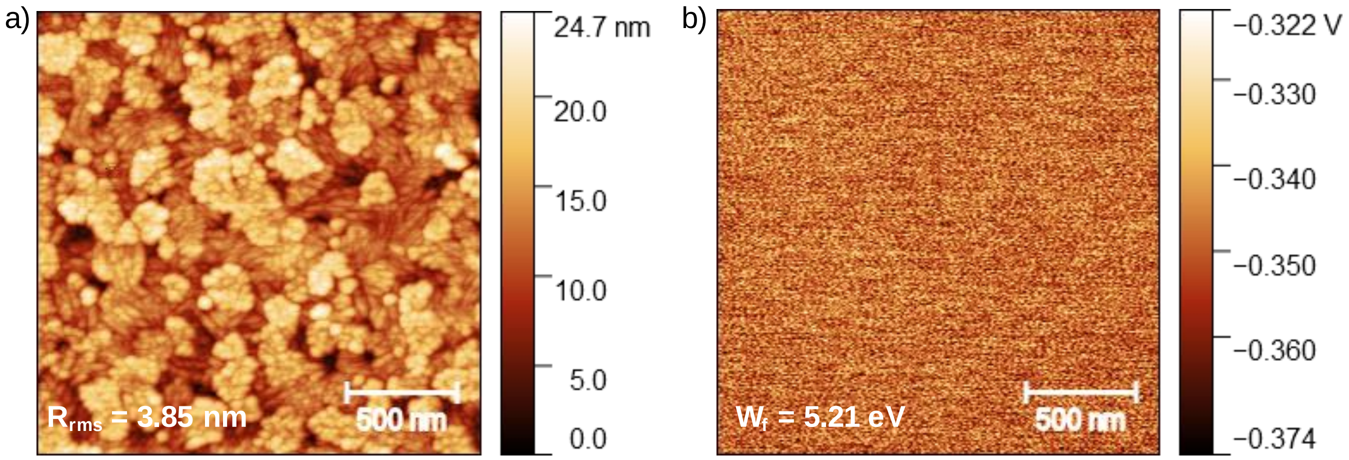

Figure S11 - a) Topography and b) surface potential of ITO/Br-2PACz substrates, obtained by AFM and Kelvin Probe method.

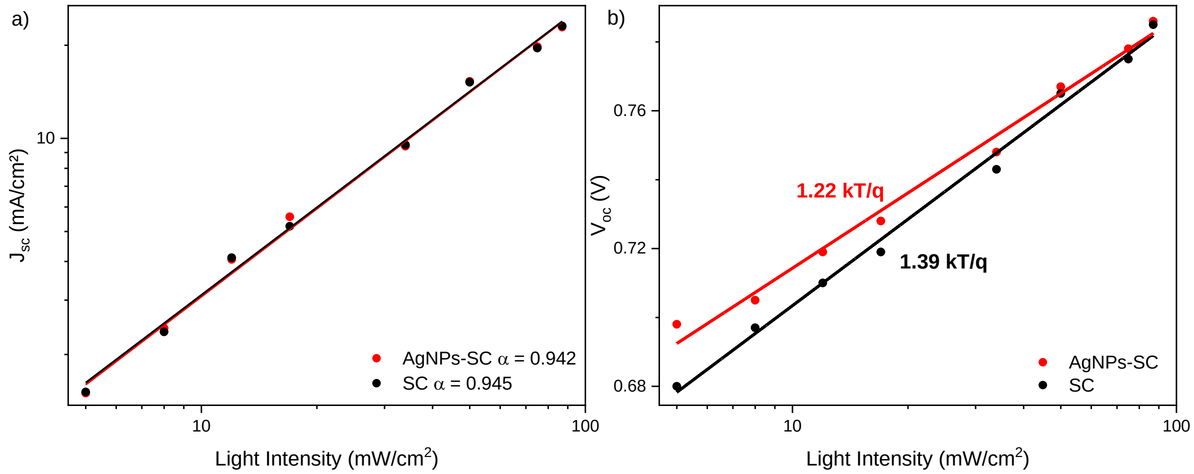

Figure S12 - Light intensity dependence of photovoltaic parameters a) J_{SC} and b) V_{OC} for SC and AgNPs-SC devices.

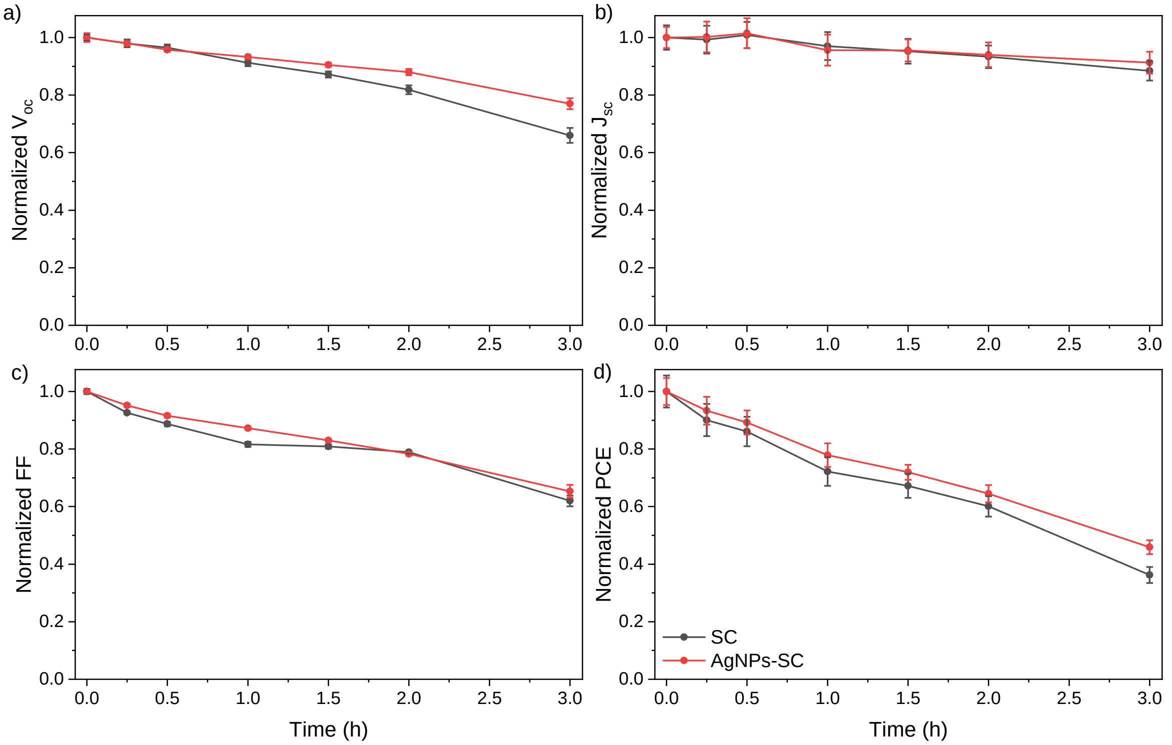

Figure S13 - Stability of organic photovoltaic devices (a) Normalized V_{oc}, b) Normalized J_{sc}, c) Normalized FF and d) Normalized PCE over Time. Under air and dark conditions with RH 65–85% over 3 hours of exposure.

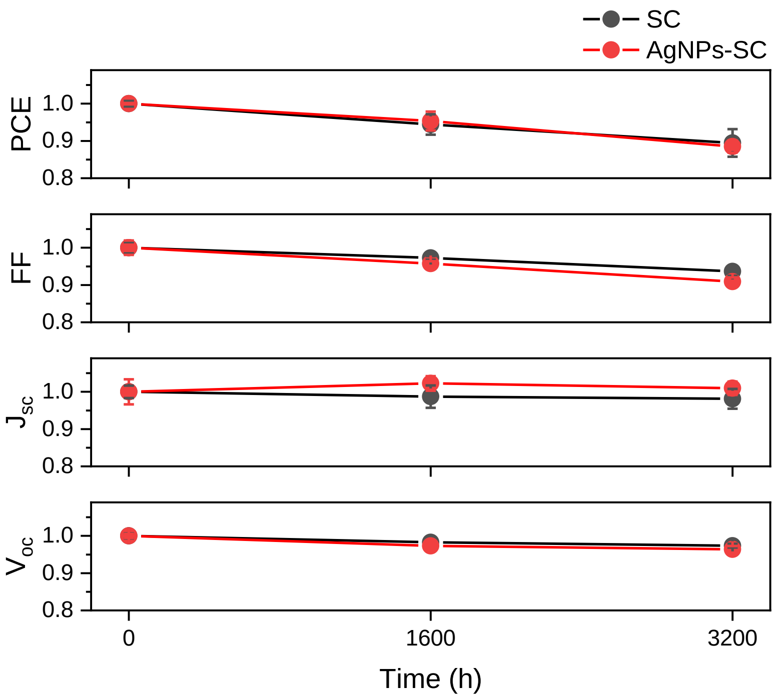

Figure S14 - Normalized Photovoltaic Parameters, of cells with and without the AgNPs layer, in fresh state (0h) and after 1600 h and 3200 h of storage in dark conditions in glovebox N2 atmosphere (~ 3 ppm of moisture and O_2).

S3 – Electrical measurements

Transient Measurements: The second-harmonic of a pulsed Nd^{3+}:YAG laser (wavelength 532 nm, pulse duration 25 ps, repetition rate 20Hz) was utilized as the pump for both transient photovoltage (TPV), transient photocurrent (TPC) measurements and Photo-CELIV. TPV measurements were conducted in the open-circuit state (impedance of 1 MΩ), while TPC measurements were carried out in the short-circuit state (impedance of 30 Ω). Data acquisition was performed using a Tektronix 3032B oscilloscope. For the Photo-CELIV measurements, an Agilent 33522A waveform generator was used in series with the sample and using the experimental setup of the previous transient measurements. In both TPV and TPC a decay of an excess of voltage is recorded in the oscilloscope (see circuit – Fig. S9) and the decay time is recorded. During these measurements the device remains illuminated by 1 sun (AMG 1.5). Similar apparatus was used for Photo-CELIV measurements, in which after the laser pulse, two consecutive voltage ramps record the electric current making use of the oscilloscope (Fig. S10).

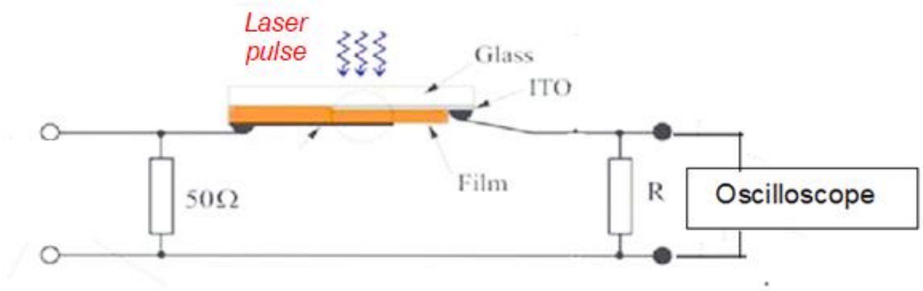

Figure S15 – TPV, TPC and Photo-CELIV experimental apparatus.

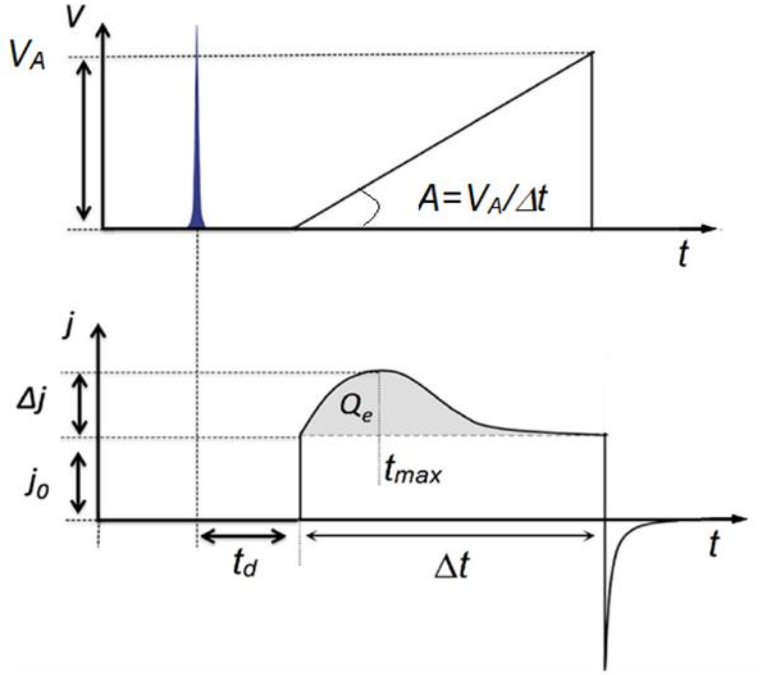

Figure S16 – Voltage ramp applied during the Photo-CELIV measurements and the scheme of the recorded electric current.

[1] R. Kerremans, C. Kaiser, W. Li, N. Zarrabi, P. Meredith, A. Armin, The Optical Constants of Solution-Processed Semiconductors—New Challenges with Perovskites and Non-Fullerene Acceptors, Adv Opt Mater 8 (2020) 2000319. https://doi.org/https://doi.org/10.1002/adom.202000319.

[2] R.C.I. MacKenzie, V.S. Balderrama, S. Schmeisser, R. Stoof, S. Greedy, J. Pallarès, L.F. Marsal, A. Chanaewa, E. von Hauff, Loss Mechanisms in High Efficiency Polymer Solar Cells, Adv Energy Mater 6 (2016) 1501742. https://doi.org/https://doi.org/10.1002/aenm.201501742.
